# Estimation of influenza‐attributable medically attended acute respiratory illness by influenza type/subtype and age, Germany, 2001/02–2014/15

**DOI:** 10.1111/irv.12434

**Published:** 2016-11-18

**Authors:** Matthias an der Heiden, Udo Buchholz

**Affiliations:** ^1^ Department of Infectious Disease EpidemiologyRobert Koch InstituteBerlinGermany

**Keywords:** burden of disease, generalised additive model, Germany, influenza, influenza type/subtype, medically attended acute respiratory illness

## Abstract

**Background:**

The total burden of influenza in primary care is difficult to assess. The case definition of medically attended “acute respiratory infection” (MAARI) in the German physician sentinel is sensitive; however, it requires modelling techniques to derive estimates of disease attributable to influenza. We aimed to examine the impact of type/subtype and age.

**Methods:**

Data on MAARI and virological results of respiratory samples (virological sentinel) were available from 2001/02 until 2014/15. We constructed a generalized additive regression model for the periodic baseline and the secular trend. The weekly number of influenza‐positive samples represented influenza activity. In a second step, we distributed the estimated influenza‐attributable MAARI (iMAARI) according to the distribution of types/subtypes in the virological sentinel.

**Results:**

Season‐specific iMAARI ranged from 0.7% to 8.9% of the population. Seasons with the strongest impact were dominated by A(H3), and iMAARI attack rate of the pandemic 2009 (A(H1)pdm09) was 4.9%. Regularly the two child age groups (0‐4 and 5‐14 years old) had the highest iMAARI attack rates reaching frequently levels up to 15%‐20%. Influenza B affected the age group of 5‐ to 14‐year‐old children substantially more than any other age group. Sensitivity analyses demonstrated both comparability and stability of the model.

**Conclusion:**

We constructed a model that is well suited to estimate the substantial impact of influenza on the primary care sector. A(H3) causes overall the greatest number of iMAARI, and influenza B has the greatest impact on school‐age children. The model may incorporate time series of other pathogens as they become available.

## 1 Introduction

The total burden of influenza in primary care is difficult to assess. Part of the challenge is that influenza cases are both frequent and may present clinically as a variety of syndromes. While the common notion is that influenza presents with fever and systemic symptoms, such as headache or myalgia, it is now well known that a large proportion of influenza disease may present also as a mild form, indistinguishable from the illness caused by other respiratory viruses.^1^ Thus, syndromic (combined with virological) surveillance systems have been put in place in many countries to monitor intensity and spread of influenza. The two major syndrome categories that have been used are acute respiratory illness (ARI) and influenza‐like illness (ILI). Among European countries, a considerable variation of case definitions has been employed for both syndromes.^2^ ILI case definitions may include the presence of fever^3^ (or another systemic symptom)^4^ in addition to one or more respiratory symptoms. In contrast, ARI case definitions usually do not require an obligatory presence of fever or feverishness.^4^ As a result, ILI case definitions are more specific, but less sensitive compared to ARI, and as a corollary surveillance systems using ILI see more pronounced illness waves and peaks during influenza epidemics. However, because only a portion of all symptomatic influenza cases are captured by ILI case definitions,^1^, ^5^, ^6^, ^7^, ^8^, ^9^ ILI surveillance systems are less well suited to describe and capture the burden of disease of influenza.

In 2013, we published results of a cyclic regression (“sin/cos‐excess model”) model that estimated the excess burden of MAARI during periods of influenza circulation compared to a baseline (using a combination of sine and cosine curves) that was established for all weeks leaving out periods of influenza circulation.^10^ We demonstrated that the season‐specific attack rate (cumulative incidence) of influenza‐attributable MAARI (iMAARI) is lower in older age groups. We demonstrated also that the order among the child age groups (aged 0‐4 and 5‐14 years, respectively) varies; that is, in some years children aged 0‐4 years had the highest attack rate, in others children aged 5‐14 years. Because we worked with ARI data, this model enabled us to estimate the total number and attack rate of iMAARI in primary care. In strong seasons, such as in 2004/05 or 2008/09, up to 9% of the general population (i.e. seven of 82 million inhabitants) were seeking health care due to an influenza infection.

In the sin/cos‐excess model, the periods of influenza circulation were determined using virological data. These data also include type and subtype information for influenza, and therefore, we intended to develop a model that allows to derive the number and the attack rate of MAARI attributable to influenza type and subtype stratified by age group. We also wanted to overcome a limitation of the sin/cos‐excess model that produced occasionally negative excesses during some weeks of a period of influenza circulation. These occurred, for example, at the end of such a period, because influenza was still confirmed in the laboratory; however, the impact on the primary care sector had waned already. Moreover, the sin/cos‐excess model could not easily be generalized to estimate the weekly number of MAARI attributable to other relevant pathogens, such as respiratory syncytial virus (RSV), human metapneumovirus (hMPV) or rhinovirus, which we have started to systematically collect only since 2013/14. The model presented in this study should be capable in the future to incorporate these upcoming data, too.

## 2 Methods

### 2.1 Data

In Germany, national surveillance for influenza on primary care level is organized by the “Working Group for Influenza” (“Arbeitsgemeinschaft Influenza” (AGI); influenza.rki.de), which collects data on medically attended ARI (MAARI).^11^ Briefly, sentinel physicians in Germany cooperate in the AGI covering approximately 1%‐1.5% of the total population of approximately 82 million persons. Physicians report aggregated age group‐specific frequencies of patients presenting with acute respiratory illness (syndromic surveillance). “Acute respiratory illness” is defined as pharyngitis, bronchitis or pneumonia with or without fever. Data are collected in the following age groups: 0‐4, 5‐14, 15‐34, 35‐59 and 60 years and older. Data are sent to and analysed by the AGI (influenza.rki.de) yielding the MAARI attack rate by age group and calendar week. Physicians record illness syndromes regardless if an individual patient had presented already earlier in the season with the same syndrome. Thus, an individual patient may contribute illness data more than once in a given season. However, a second occurrence of an illness of an individual patient is only recorded again if at least 2 weeks have passed after the first incident.^10^ In addition, a subset of about 20% of the sentinel physicians collects respiratory samples from patients with influenza‐like illness (ILI) which are sent to the National Reference Center for influenza (NRCI). During the study period, physicians participating in the virological surveillance arm were requested to take respiratory samples from patients presenting with influenza‐like illness. “Influenza‐like illness” was defined as acute respiratory illness with fever and cough or sore throat. Physicians were asked to take at least one, but not more than two samples from a given age group (0‐4 years, 5‐14, 15‐34, 35‐59 and 60+ years). All samples were tested and typed in the NRCI by real‐time PCR for presence of influenza A, B and the subtypes A(H1N1) and A(H3N2).^10^ For the study, we used data from the syndromic and virological surveillance arm of the AGI comprising the period 2001/02 through 2014/15, for a total of 14 seasons, including the pandemic 2009/2010.

### 2.2 GAM sample‐based Model

We used the age‐specific weekly MAARI attack rate *m*_*t*_ as dependent variable and developed a generalized additive regression model^12^ with linear link function to analyse this curve. The model was stratified with respect to age group; to simplify the notation, we omitted in the formulas the subscript for age group. We denoted by *i*_*t*_ the (age group‐specific) number of respiratory samples with ILI having tested positive for influenza in week *t*. From hereon, we refer to *i*_*t*_ as the course of laboratory‐confirmed influenza. We made the following assumptions in each age group:

1. The MAARI attack rate *m*_*t*_ can be described as additive composition of a periodic baseline, a secular trend and the iMAARI attack rate.
2. In each season, the course of the laboratory‐confirmed influenza, *i*_*t*_, mirrors the course of the attack rate of influenza‐attributable ILI (iMAILI) in the total population.
3. In each season, the age group‐specific proportion of iMAILI among the iMAARI is approximately constant over the weeks.

We modelled the periodic MAARI baseline using a penalized cyclic p‐spline *f*(*p*_*t*_) with at most 52 knots—one for each calendar week; here, *p*_*t*_ counts the calendar weeks of the year. The secular trend was modelled by a penalized p‐spline *g*(*t*) with at most 7 knots (1 for every two seasons). The third component of the model is *i*_*t*_ multiplied by a season‐specific factor $\beta_{s_{t}}$, where *s*_*t*_ describes the season week *t* belongs to. The formula of the expected weekly MAARI attack rate for each age group (five models) then looks as follows:

(1) $$E\left(m_{t}\right)=f\left(p_{t}\right)+g\left(t\right)+\beta_{s_{t}}i_{t}.$$

Hence, we estimated the expected iMAARI attack rate in week *t* as

(2) $$E\left({\hat{e}}_{t}\right)={\hat{\beta }}_{s_{t}}i_{t}.$$

When this model is used prospectively, the inclusion of *i*_*t*_ with a season‐specific factor is only useful after the start of a period of influenza circulation, for example as defined in an der Heiden,^10^ after the lower confidence limit of the proportion of samples positive for influenza in the NRCI exceeds 10% in two consecutive weeks. A potential season without a period of influenza circulation should be disregarded.

In weak seasons, the estimated coefficient ${\hat{\beta }}_{s_{t}}$, of a particular age group might be a negative number, as the observed MAARI may lie beyond the baseline. In this case, the expected iMAARI attack rate in this age group will be negative throughout the season. We conclude then, that it cannot be quantified and put it to zero.

**Figure 1:**
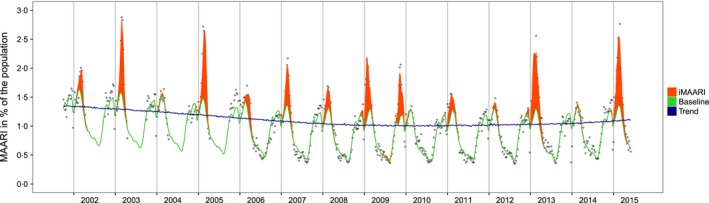
Raw data (medically attended acute respiratory illness (MAARI) in per cent of the population (hollow dots), data modelled by the GAM sample‐based model (baseline; green line), secular trend (blue line), MAARI attributed to influenza (iMAARI; red‐shaded area). Vertical lines represent the change of the year, Germany, 2001/2002–2014/2015

In a second step, we subdivided the estimated iMAARI attack rate according to the distribution of types and subtypes, A(H1), A(H3) or B (Figure 1, top), in the respiratory samples. A(H1) before the pandemic 2009 was named A(H1)prepan, and A(H1) from 2009 onwards, the year of the pandemic, was named A(H1)pdm09. To obtain a more stable estimate of the subtype distribution *d*_*t*_ of week *t*, we used the subtype information of all‐influenza‐positive specimens tested in weeks *t*−1, *t* and *t* + 1. We made the following additional assumption:

The (age‐specific) distribution *d*_*t*_ of influenza subtypes describes the distribution of samples among iMAILI patients in the total population and is a valid proxy for the distribution of samples among iMAARI patients.

We combined the uncertainties of the estimated iMAARI and the (sub)type distribution by drawing random samples from the model normal distribution $N\left(\beta_{s_{t}},{\sigma_{\beta_{s_{t}}}}^{2}\right)i_{t}$ and the Dirichlet distribution ${\hat{d}}_{t}=D\left(n_{t}\left(H1\mathrm{pdm}09\right),n_{t}\left(H1\mathrm{prepan}\right),n_{t}\left(H3\right),n_{t}\left(B\right)\right)$. The latter describes the uncertainty in the (sub)type distribution; *n*_*t*_ is the number of samples having tested positive for the respective (sub)type in week *t* and its two neighbouring weeks.

The expected iMAARI attack rate associated with influenza of subtype *τ* is then given by

(3) $$E\left({\hat{e}}_{t,\tau }\right)=E\left({\hat{e}}_{t}\right)\cdot {\hat{d}}_{t}\left(\tau \right).$$

Based on the consideration that a high influenza attack rate in one season may lead to a relative immunity of at least 1 year (until the virus has drifted sufficiently to evade immunity), one could postulate that a season with a higher attack rate (overall, or in a particular type/subtype) may be followed by a lower attack rate and vice versa. We therefore attempted to “predict” the magnitude of iMAARI attack rates (by type/subtype and overall) based on the magnitude of the preceding season. To do that, we built thirteen pairs with the iMAARI attack rate of the season of interest as dependent variable and that of the preceding season as explanatory variable (the first season (2001/02) had no preceding season and dropped out). We used a Poisson regression to quantify the associations between the pairs and checked both qualitatively and with pseudo‐*R* ^2^ how well these considerations were met by the data.

### 2.3 Sensitivity analysis

S1: To check the stability of our model, we modified the number of seasons included in our model (Equation (1)). We estimated the iMAARI season attack rate starting with the use of the entire history of seasons, that is from 2001/02. We then applied the GAM sample‐based model to data sets that consecutively omitted historic seasons beginning with the most distant ones first until only five seasons of data remained. Thus, for the seasons 2010/11 to 2014/15 ten different estimates were calculated. The resulting estimates for the iMAARI attack rate were compared for all age groups combined and also separately for the five age groups used in this study.

S2: To gauge the effect of varying degrees of freedom in the secular trend, we also considered a model with a more flexible secular trend that allowed 1 *df* per included season, that is 14 *df* instead of 7. We estimated the iMAARI attack rates in an ongoing season simulating from retrospective data that additional information becomes available as the season is evolving. We used season 2013/14 as example and calculated (cumulative) iMAARI attack rates for all age groups combined and for the five age groups separately.

S3: To understand better the differences of our previous (sin/cos‐excess) model^10^ to the GAM sample‐based model presented in this study, we compared iMAARI attack rates derived from these two models with each other. Moreover, we considered an intermediate model “GAM‐excess” model that incorporates characteristics of both the sin/cos‐excess model and the GAM sample‐based model. It leaves out periods of influenza circulation for model building (as done in the sin/cos‐excess model^10^) but uses penalized p‐splines for the secular trend and the cyclic component (as in the GAM sample‐based model).

All estimations including the fitting of generalized additive models (package “mgcv”) were performed using version 3.3.0 of the statistical analysis software r.^13^

## 3 Results

### 3.1 Model fit

The GAM sample‐based model (described by Equation (1)) showed a good fit to the data in various aspects (Figure 1). The model had an adjusted *R* ^2^ value of 97.4%, and 98.8% of the deviance in the data could be explained by the model. We found a decreasing secular trend that stabilized after 2009. The period pattern showed increased MAARI activity (without influenza) in autumn and throughout the winter. After periods of influenza circulation (red‐shaded areas in Figure 1), MAARI activity declined and reached the annual lowest points around the middle of the year. Troughs can be seen during the weeks around the change of the year and smaller troughs during weeks around the autumn holidays in Germany. Nevertheless, in several seasons, for example during the years 2002, 2003 and 2005, there is MAARI activity in the autumn in addition to that captured by the model.

### 3.2 Virological data

**Figure 2:**
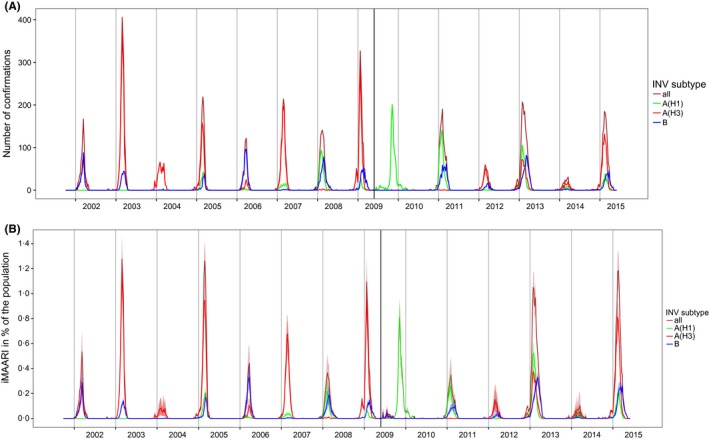
Top panel: number of influenza confirmations by type and subtype among sentinel respiratory samples, by season. Bottom panel: type‐ and subtype‐specific influenza‐attributable medically attended acute respiratory illnesses (iMAARI), in % of the population, by season. The extra vertical line indicates the beginning of the pandemic A(H1)pdm09. A(H1)prepan=pre‐pandemic A(H1)

The frequency and proportional distribution of (sub)types varied considerably from season to season (Figure 2, top panel; Table 1). Until 2009 (the pandemic season), A(H1)prepan, A(H3) and B cocirculated, albeit not in every season to the same degree. After the advent of A(H1)pdm09 in 2009, circulation of A(H1)prepan ceased, and A(H3) and B paused, but continued to cocirculate together with A(H1)pdm09 from 2010/11. Seasons where only one type or subtype dominated almost exclusively were rare and occurred only in 2003/04 (A(H3)) and during the pandemic 2009/10 (A(H1)pdm09). All other seasons experienced some degree of simultaneous and overlapping circulation of two or three types or subtypes. However, only in some seasons, such as in 2001/02 and 2005/06 peaks of types or subtypes occurred at the same time, and were desynchronized otherwise.

**Table 1:** Frequency distribution of the number of respiratory specimens positive for influenza taken by sentinel physicians, as well as estimated number of patients and proportion of the population with medically attended acute respiratory infections (MAARI) due to influenza (iMAARI)

Season	Number of samples	Influenza‐positive samples	Number of iMAARI (in million)	95% confidence interval	iMAARI attack rate in %	95% confidence interval
2001/02	2935	802	2.1	(1.5‐2.7)	2.5	(1.8‐3.3)
2002/03	4376	2000	5	(4.4‐5.6)	6.1	(5.3‐6.8)
2003/04	2831	583	0.6	(0.2‐1.2)	0.7	(0.2‐1.5)
2004/05	3792	1171	5.5	(4.9‐6.2)	6.7	(5.9‐7.5)
2005/06	1934	632	1.9	(1.3‐2.5)	2.3	(1.6‐3.0)
2006/07	2841	1245	3.3	(2.6‐4.0)	4	(3.2‐4.9)
2007/08	2513	1101	2.2	(1.4‐3.0)	2.7	(1.8‐3.6)
2008/09	3416	1698	4.6	(3.9‐5.4)	5.6	(4.8‐6.6)
2009/10	3526	1179	4	(3.3‐4.8)	4.9	(4.0‐5.9)
2010/11	2950	1419	2.1	(1.4‐2.9)	2.6	(1.8‐3.6)
2011/12	1789	411	0.8	(0.4‐1.5)	1	(0.5‐1.8)
2012/13	3961	1840	7.2	(6.3‐8.0)	8.9	(7.8‐9.9)
2013/14	2290	243	0.6	(0.2‐1.2)	0.8	(0.3‐1.5)
2014/15	3934	1461	7.1	(6.2‐8.0)	8.7	(7.6‐9.9)

### 3.3 Influenza‐attributable MAARI

In most seasons, we found a considerable amount of MAARI explained by circulation of influenza viruses (iMAARI; red‐shaded areas in Figure 1) with a wide variation among seasons (Figure 2, bottom panel; Table 1). The proportion of the population with iMAARI ranged from 0.74% in 2003/04 (0.6 million individuals) to 8.9% in season 2012/13 (7.2 million individuals; Table 1). In the median influenza season, 3.4% of the population (2.8 million individuals) consulted a physician due to their influenza infection (interquartile range, 2.3%‐6.0% (1.9‐4.9 million individuals)). The pandemic in 2009 led to an iMAARI attack rate of 4.9% (4.0 million persons) of the population, but is surpassed in magnitude during the time period analysed (2001‐2015) by five non‐pandemic seasons.

The ratio of the number of confirmed influenza specimens (Figure 2, top panel) to the iMAARI attack rate (Figure 2, bottom) differed among seasons, showing the necessity of a season‐dependent factor in Equation (1). For example, in season 2002/03 the number of influenza‐positive samples was substantially higher than in season 2004/05, whereas the estimated number of iMAARI was higher in 2004/05 (Figure 2; Table 1 (column 4)).

**Figure 3:**
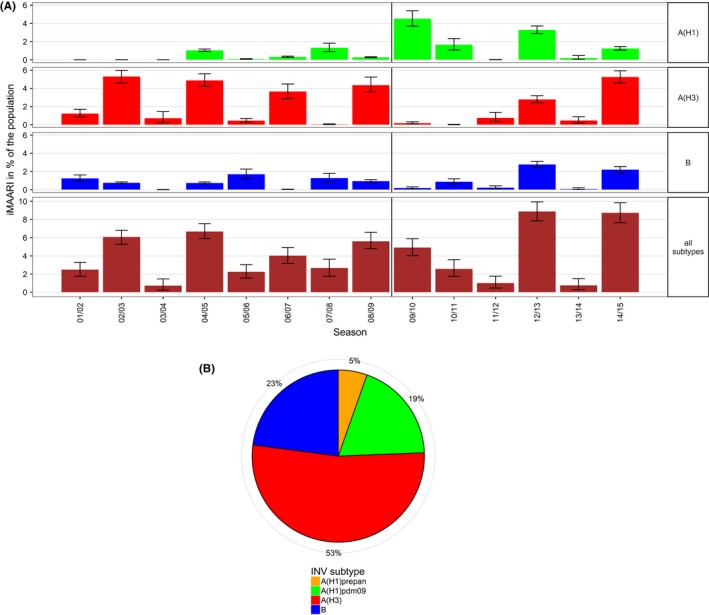
Top panel: estimated number of influenza‐attributable medically attended acute respiratory infections (iMAARI) by influenza type/subtype and season; green=A(H1) (pre‐pandemic A(H1) (A(H1)prepan) and A(H1)pdm09 not separated), red=A(H3), blue=B. Bottom panel: distribution of all iMAARI accumulated for all seasons from 2001/02 until 2014/15, by type/subtype. Colours denote influenza types and subtypes; in contrast to the top panel, A(H1)prepan (orange) and A(H1)pdm09 (green) are coloured separately

A somewhat alternating pattern of stronger and weaker seasons between 2001/02 and 2008/09 was interrupted by the pandemic 2009. However, since 2011/12 this pattern seems to have picked up again (Figure 2); among the types and subtypes, this pattern can be observed only for A(H3) (Figure 3, top panel). In general, seasons with the strongest impact (2002/03, 2004/05, 2008/09, 2012/13 and 2014/15) were dominated by A(H3), except for 2012/13, where also A(H1)pdm09 and B cocirculated and led to iMAARI to a similar degree as A(H3) (Figure 2, bottom panel; Table 2; Figure 3). Overall, there were seven (sub)type–seasons where a type or subtype led to at least 3% iMAARI in the population. Five (71%) of these were caused by A(H3), two (29%) by A(H1)pdm09, none by A(H1)prepan and none by influenza B. On the other hand, dominance of A(H3) did not always lead to strong seasons: also some of the weakest seasons (2003/04, 2011/12 and 2013/14) were dominated by A(H3). During the 14 seasons and among the three types and subtypes, A(H3) led to the highest iMAARI attack rate in eight (57%) of the seasons, A(H1) in four (29%) and B in two (14%). Except in 2003/04, at least two (sub)types led to iMAARI attack of at least 0.2% of the population (150 000 individuals; Table 2); in only one season (2013/14), no subtype led to an iMAARI attack rate of more than 0.5% (Table 2; 400 000 individuals); and in only one season (2012/13), all three subtypes led to an iMAARI rate of more than 2.5% of the population.

**Table 2:** Number of A(H1)‐, A(H3)‐ and B‐positive respiratory samples collected by sentinel physicians in each season and estimated attack rate of medically attended acute respiratory infections due to influenza types and subtypes (iMAARI)

Season	A(H1)	A(H3)	B	A(N1)		A(H‐3)		B	
				iMAARI attack rate in %	95% confidence interval	iMAARI attack rate, in %	95% confidence interval	iMAARI attack rate, in%	95% confidence interval
2001/02	0	417	385	0.0	(0.0‐0.1)	1.2	(0.8‐1.7)	1.3	(0.9‐1.6)
2002/03	1	1720	279	0.0	(0.0‐0.1)	5.3	(4.6‐6.0)	0.8	(0.7‐0.9)
2003/04	0	578	5	0.0	(0.0‐ 0.1)	0.7	(0.2‐0.5)	0.0	(0.0‐0.1)
2004/05	215	806	150	1.1	(0.9‐1.2)	4.9	(4.2‐5.6)	0.8	(0.7‐0.9)
2005/06	22	102	508	0.1	(0.1‐0.1)	0.5	(0.3‐0.7)	1.7	(1.2‐2.3)
2006/07	124	1110	11	0.3	(0.3‐0.4)	3.7	(2.9‐4.5)	0.0	(0.0‐0.1)
2007/08	572	15	514	1.3	(0.9‐1.8)	0.1	(0.0‐0.1)	1.3	(0.8‐1.8)
2008/09	104	1226	368	0.3	(0.2‐0.3)	4.4	(3.6‐5.2)	1.0	(0.8‐1.1)
2009/10	1174	2	3	4.5	(3.7‐5.4)	0.2	(0.1‐0.3)	0.2	(0.1‐0.3)
2010/11	882	9	528	1.7	(1.1‐2.3)	0.0	(0.0‐0.1)	0.9	(0.6‐1.2)
2011/12	4	9	97	0.0	(0.0‐0.1)	0.8	(0.4‐1.4)	0.2	(0.1‐0.4)
2012/13	618	310	647	3.3	(2.9‐3.7)	2.8	(2.4‐3.2)	2.8	(2.5‐3.1)
2013/14	73	148	22	0.2	(0.1‐0.5)	0.5	(0.2‐0.9)	0.1	(0.0‐0.2)
2014/15	219	910	332	1.3	(1.1‐1.5)	5.3	(4.6‐5.9)	2.2	(1.9‐2.5)

Summed up over all 14 seasons, a total of 48.2 million physician visits occurred by patients with influenza. The majority of iMAARI (53%) was caused by A(H3) followed by influenza B (23%), A(H1)pdm09 (19%) and A(H1)prepan (5%; Figure 3, right panel). Both A(H1) subtypes together were responsible for roughly a quarter of all iMAARI in the total study period.

**Figure 4:**
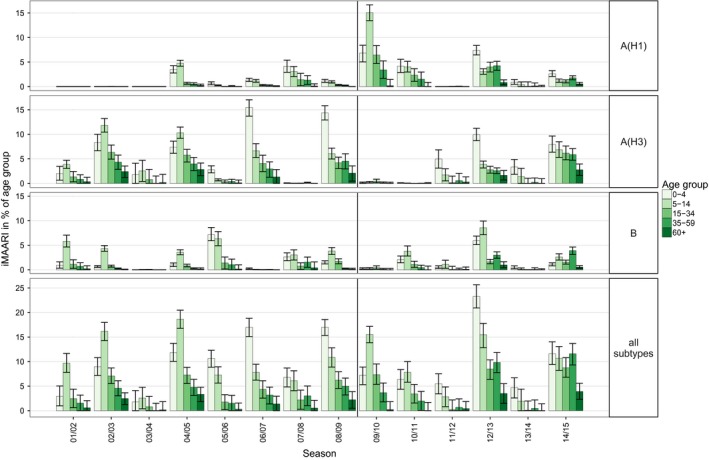
Age‐ and season‐specific attack rate of influenza‐attributable medically attended acute respiratory illnesses (iMAARI), in % of age group

Seasonal iMAARI attack rates varied by age (Figure 4). For all three types and subtypes and in all seasons, one or both of the two child age groups (0‐4, 5‐14) had the highest iMAARI attack rates, with only one exception. For influenza B, in 2014/15 the 35‐ to 59‐year‐old age group had the highest attack rate. Generally, after the two child age groups the age‐specific iMAARI attack rate decreased with increasing age. In eight (57%) of 14 seasons, at least one of the two child age groups had an attack rate of more than 10%. In the pandemic season 2009/10, the age group 5‐14 was by far the most affected and had an iMAARI attack rate that was more than two times higher than either the 0‐ to 4‐ or the 15‐ to 34‐year‐old age group.

**Figure 5:**
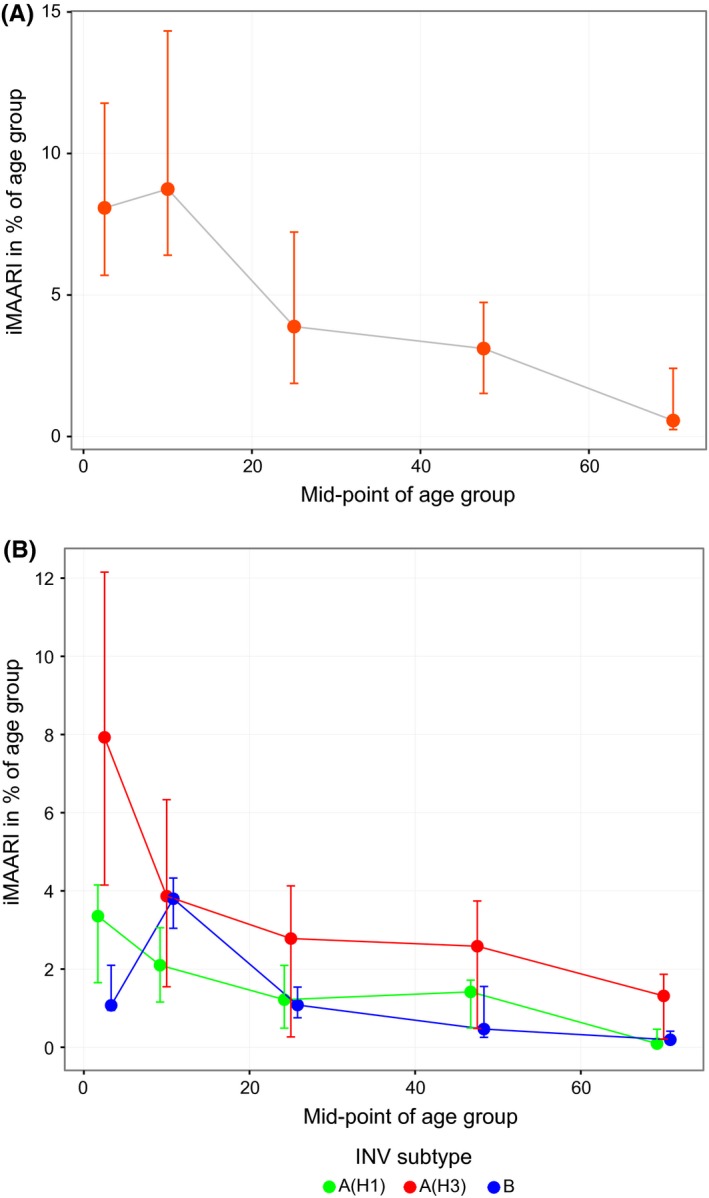
Top panel: attack rates of influenza‐attributable medically attended acute respiratory infections (iMAARI), median of 14 seasons (2001/02‐2014/15), by age (shown are mid‐points of five age groups). Bottom panel: age‐specific median and interquartile range of iMAARI attack rates across all seasons with typical pattern stratified by type/subtype. Points for the medians were connected by lines to guide the eye

Figure 5 (top panel) sums up the age‐ and season‐specific all‐influenza iMAARI attack rates displaying the median and the interquartile range of all 14 seasons. The median seasonal attack rates were approximately 9% in both child age groups and 4% among the 15‐ to 34‐year‐old age group and went down to approximately 0.5% among the 60+.

In Table 3, we categorized the age patterns of the season attack rates into three classes: (i) “low” meaning that all age‐specific attack rates in a season were below 1%; (ii) “monotone” means the pattern was not low and the age group 0‐4 had the highest attack rate, that is generally decreasing to that of age group 60+, that had the smallest attack rate or was below 0.5%; and (iii) “skewed hat” means the season was not low and the age group 5‐14 had the highest attack rate, that is generally decreasing to that of age group 60+ that had the smallest attack rate or was below 0.5%. The low, monotone and skewed‐hat patterns are shown as icons in Table 3. The typical pattern was “monotone” for A(H1) and A(H3) and “skewed hat” for B.

**Table 3:** Number of seasons with a particular pattern of the attack rates over the age groups

	Pattern of age group‐specific attack rates		
	Monotone	Skewed hat	Low
	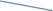	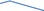	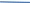
INV subtype			
A(H1)	6	2	6
A(H3)	7	4	3
B	1	9	4

In the right panel of Figure 5, the age‐specific iMAARI attack rates are summed up by (sub)type for those seasons that followed the typical pattern. The median ratio of the iMAARI attack rates of the age group 0‐4 to the age group 5‐14 was 1.28 (IQR: 1.17‐2.02) for influenza A(H1) and 2.43 (IQR: 2.36‐2.73) for influenza A(H3). In contrast, for influenza B this ratio was 0.41 (IQR: 0.28‐0.55).

**Figure 6:**
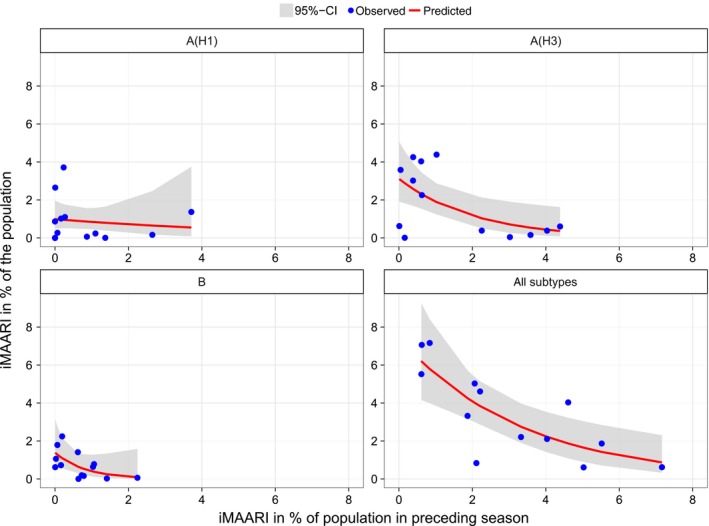
Observed and predicted iMAARI attack rate based on iMAARI attack rate in the preceding season. Dots show iMAARI attack rates as observed, and lines show modelled iMAARI attack rates as predicted using Poisson regression

All‐influenza iMAARI attack rates of a season predicted the iMAARI attack rate in the following season relatively well; strong seasons were followed by weaker ones and vice versa (Figure 6). McFadden´s pseudo‐R² has a value of 61%, showing that most of the variation can be explained by the Poisson model. For A(H3) and B, the model fitted data less good, and even worse for A(H1); this corresponds to pseudo‐R² of 36% and 39% for A(H3) and B, respectively, and only 1.9% for A(H1). For A(H3), the data points group in two locations, either on a low level or on a higher level, representing the up‐ and down‐rhythm of A(H3) seasons. In other words, a strong A(H3) season is followed by a weak A(H3) season (less than 1% of the population), whereas a weak A(H3) season is less narrowly predictive. The two dots on the A(H3) graph in the lower left corner represent the first and second season after the pandemic season.

### 3.4 Sensitivity analysis

S1: Using our GAM sample‐based model on different data sets and comparing the estimates for the included seasons showed that the estimated iMAARI attack rate in a given season depends on the whole history of data included (Fig. S1A). Nevertheless, most changes lie inside the range given by the 95% confidence intervals. Moreover, the differences within seasons are substantially smaller than the differences between seasons; hence, the ranking of the seasons regarding the iMAARI attack rate was quite stable. Considering only seasons after the pandemic season 2009/10 leads to systematically higher estimates for the iMAARI attack rates. These are due to deviations in the two child age groups (Fig. S1B). In contrast, estimates for the age groups 15‐34, 35‐59 and 60+ demonstrated remarkable stability independently of the history of seasons included in the models.

S2: Using a GAM sample‐based model with a more flexible secular trend, that is 1 *df* per one instead of two included seasons, led to less stability regarding the estimated iMAARI attack rate in the current season. We demonstrate the estimates in the course of season 2013/14 as an example (Fig. S2A). In particular, in calendar week 11/2014 the estimate was too high and had to be corrected downwards thereafter. In addition, iMAARI of the age group 0‐4 was quite instable as it seemed to overshoot not only in the latter part of the season but also very early on (Fig. S2D). In comparison, the model using the less flexible secular trend resulted in more stable estimates (Fig. S2C). This confirmed our choice of a relatively inflexible trend with 7 *df*.

S3: Comparing the estimated iMAARI season attack rates showed that the intermediate (GAM‐excess) model and the GAM sample‐based model agreed by and large, whereas the previous (sin/cos‐excess) model^10^ led in several seasons to noticeably higher estimates. The one exception is the pandemic (Fig. S3A). This relates to the fact that the baseline in the sin/cos‐excess model showed in most seasons a bimodal course with two peaks of similar height separated by the turn of the year (Fig. S3B). The GAM sample‐based model, however, has a lower peak before the turn of the year and a higher peak in January/February. As the influenza epidemic almost always starts after the turn of the year, the resulting excess of MAARI is therefore higher in the sin/cos‐excess model. Again the exception of this rule is the 2009 pandemic which occurred in October/November. The sin/cos‐excess model calculates a lower number of iMAARI for the 2009 pandemic compared to the GAM sample‐based model.

In the season 2005/06, the baseline of the sin/cos‐excess model^10^ (Fig. S3B) during the period of influenza circulation is very similar to that of the intermediate (GAM‐excess) and GAM sample‐based model resulting in similar iMAARI estimates (Fig. S3A). However, in the season 2006/07 the lower pre‐influenza MAARI rates “pull” the baseline during the period of influenza circulation somewhat down (circle in 2006/07 in Fig. S3B) resulting in higher iMAARI estimates compared to those generated by the intermediate (GAM‐excess) and GAM sample‐based model (Fig. S3A).

## 4 Discussion

We have developed a model that uses virological type and subtype as well as age specific data from sentinel physician surveillance to “explain” MAARI attributable to influenza using a GAM regression model. Output from this model includes estimates of consultation burden on primary care level stratified by influenza type, subtype and age.

This type of model is warranted because we have collected ARI consultations which have a high background rate and are a more non‐specific indicator for influenza than ILI. On the other hand, because of the sensitive case definition (ARI), it has the advantage that burden estimations (with the aim to calculate disability‐adjusted life‐years (DALY)) on primary care level need no further multiplier but can be calculated directly.

Compared to data from other countries that are using ILI data to estimate burden of influenza on primary care level, our estimates tend to be substantially higher. Paget has estimated the burden of paediatric influenza in four countries (England, the Netherlands, Spain and Italy) in the seasons 2002/03–2007/08.^14^ In the first three countries, the estimated season iMAILI attack rate yielded average estimates in the range of 0.35%‐2.16% for the age group 0‐4 and 0.32%‐2.96% in the age group 5‐14. Only estimates from Italy (8.9% and 9.8%) were in a comparable range to ours, but seem to represent an outlier among countries that collect ILI data. Population‐based studies have shown that the actual burden of influenza in children is under‐recognized with common (ILI) surveillance methods. For example, between 2004/05 and 2008/09 Poehling conducted a population‐based study in three US counties and found that per season, between 10% and 25% of children aged 0‐4 years sought outpatient medical care because of influenza.^15^ Our estimates in these two age groups between the seasons 2002/03 and 2008/09 ranged from 2% to 17% (age group 0‐4; median 11%) and from 3% to 13% (age group 5‐14; median 8%), respectively, and are therefore comparable with the thoroughly conducted population‐based research study. Similarly, in the three seasons following the pandemic, influenza‐associated consultations by patients with ILI were estimated in a population‐based surveillance project in 13 US health jurisdictions as 0.7%, 0.2% and 1.1%.^16^ Given that only between 30% and 80% of all influenza cases manifest themselves as ILI^5^, ^6^, ^7^, ^8^ and in the same year influenza seasons may be quite different in different countries, the estimated 2.6%, 1.0% and 8.9% in our study lie in a comparable magnitude as the US data. We believe that our combination of surveillance (using ARI data) followed by modelling estimates the population impact of influenza more realistically than sentinel systems that use ILI data. Moreover, the surveillance system used provides such estimates not just for a few counties for a limited number of seasons, as is the case for research studies, but for the entire country, the entire age range and every season.

Surveillance systems that collect ILI data may estimate iMAILI by multiplying the proportion positive for influenza among ILI patients in virological surveillance by the number of ILI patients in the population.^19^ We could not use this approach because syndromic surveillance collects data on MAARI, whereas virological surveillance focusses on ILI patients. Certainly, the distribution of respiratory viruses among all MAARI patients is not the same as for MAILI patients. For example, the influenza positivity rate among ARI is considerably lower than for ILI. Hence, in our model we do not use the proportion positive for influenza, but we used the total number of samples that tested positive for influenza, and if yes, for which type and subtype. We still have to assume that the proportion of iMAILI among iMAARI (or the iMAILI to iMAARI ratio) is constant during a given season and does not depend on the influenza type or subtype. The fact that the GAM‐excess model and the GAM sample‐based model estimated a quite similar excess in most seasons shows that the course of the laboratory‐confirmed influenza is suitable as a proxy for the iMAARI attack rate, as the GAM‐excess model does not imply any assumptions on the shape of this course. In fact, we found an almost identical baseline in both models. In seasons (07/08, 09/10, 10/11), where we found differences in the iMAARI attack rate, these resulted from weeks at the beginning or end of the influenza season. Either the observed MAARI were below the baseline and hence the GAM sample‐based model did not follow them (seasons 09/10 and 10/11) or there were additional spikes (season 07/08) that were not paralleled in the course of the laboratory‐confirmed influenza (Fig. S4). As the number of samples with laboratory‐confirmed influenza was quite low for these periods, these deviations in the observed MAARI rate were rather not directly connected to influenza.

The previous model^10^ used sine/cosine curves to calculate periodicity of the baseline. However, recently the use of splines has proved to provide a more flexible tool to estimate irregular periodic curves.^20^ Using 52 knots for the yearly oscillating baseline allowed us to construct a quite realistic pattern. A particular characteristic of the sin/cos‐excess model was that we accounted for oscillations of the MAARI baseline with periods of 2 and more years. This allowed us to adapt quite well to the MAARI we observed before the period of influenza circulation. As described in the 3 section, the GAM sample‐based model does not have this property and we observe unusual MAARI activity in the autumn period of several seasons. On the other hand, in the sin/cos‐excess model we implicitly assumed that the (sometimes) unusual autumn MAARI activity continues into the period of influenza circulation which is also not always plausible. In the end, to adequately account for unusual MAARI activity in autumn, data about the MAARI or MAILI activity due to other pathogens are needed. These are now being collected but comprehensively only since 2013/14. Until we are able to construct a stable baseline over several years, we will be using a more parsimonious model.

Due to the fact that we have estimated the total number of iMAARI over a long time frame, we were also able to cumulate these over time. While A(H3) has been associated with both very strong and quite weak influenza seasons, it has overall led to more than half of all iMAARI in those 14 seasons. In contrast, A(H1)prepan has contributed the least, first of course because it ceased to circulate after the advent of A(H1)pdm09, and second because it was generally associated with a weaker seasonal impact when it did circulate (Figure 3; Table 1). Even both A(H1) variants (pre‐pandemic and pdm09) together led only to approximately one‐quarter of all iMAARI between 2001/02 and 2014/15.

The age dependency of influenza can be seen nicely also in Figures 4 and 5. The two child age groups mostly have a rather high attack rate, then it drops and stays rather constant in “younger” adulthood (15‐59 years) before it drops again in old age. Several other population‐based studies or analyses of surveillance data have observed that medically attended respiratory illnesses on primary care level decline with age.^1^, ^21^, ^22^, ^23^, ^24^ What this study adds is the additional information of the comprehensive burden of all‐influenza cases in primary care, be they mild or more severe, not only by age, but also by type/subtype, over a long time period. It is interesting that the “typical” pattern of influenza B shows a substantially higher relative iMAARI attack rate among school‐age children^5^, ^6^, ^7^, ^8^, ^9^, ^10^, ^11^, ^12^, ^13^, ^14^ compared to that in age group 0‐4 (Figure 5, right panel), although in absolute terms the attack rate among 5‐ to 14‐year‐old children is comparable to that caused by A(H3N2). This striking characteristic of influenza B concurs with data from two serological studies, one from the Netherlands and one from Germany, which investigated the seroprevalence of antibodies against influenza virus types and subtypes by year of age among children.^17^, ^18^ Both studies showed that seroprevalence of antibodies against influenza A viruses rises faster in early childhood compared to influenza B. In the German study, seroprevalence of antibodies against influenza A rises asymptotically with age reaching a rate of approximately 90% by the age of 6‐7 years. However, seroprevalence rises only linearly for influenza B, and at the age of 6‐7 years, 70% of children are still lacking detectable IgG antibodies.^18^

Another interesting outcome of our analysis is that the impact of seasons tends to almost oscillate biannually; that is, a strong season is followed by a weaker season (Figure 2). The pandemic only interrupted this pattern for a couple of years. Indeed, when we modelled the iMAARI attack rate as an (inverse) function of the magnitude of the preceding season, we found a pattern that seems to support this observation (Figure 6). However, this is by and large influenced by the dynamics of A(H3) and less so by influenza B. The reason for this pattern may be that the degree of population immunity after a heavy season is large enough to dampen the impact of next season's influenza virus, independently of its type or subtype.

We have to admit the following limitations. First, the weekly number of samples taken for virological surveillance is somewhat capped because physicians are requested to take no more than three to five specimens per week. It is expected that this might overemphasize a little bit the tail ends of the epidemic, and it requires a season‐specific factor to calculate iMAARI from the number of confirmed influenza viruses. Second, as we are lacking a time series of data on RSV (and other respiratory pathogens) and as RSV seasons may overlap with influenza epidemics,^25^ we might overestimate the burden of influenza to a certain extent. However, data from the above mentioned study in four European countries found that in two of the four countries, RSV was not a significant term in the model (explaining MAILI), and in two others, the model attributed only 11% and 13%, respectively, to RSV.^14^ We therefore do not believe that we vastly overestimate the seasonal attack rate of iMAARI by neglecting RSV circulation.

## 5 Conclusion

We present a GAM model that is capable of estimating the influenza‐attributable MAARI attack rate on the basis of aggregated ARI as well as virological data stemming from a sentinel physician network. The model has been capable to yield type‐ and subtype‐ as well as age‐specific estimates of the burden of influenza on primary care level. The estimated seasonal iMAARI attack rate is substantially higher than in other countries using ILI surveillance data and agrees better with detailed population‐based research studies. About half of the impact during these 14 seasons was caused by A(H3). Regularly the two child age groups (0‐4, 5‐14) had the highest iMAARI attack rates reaching frequently levels up to 15%‐20%. Influenza B has led to an exceptionally high impact among 5‐ to 14‐year‐old children, compared to all other age groups. The degree of influenza activity in 1 year seems to influence the degree in the next, largely influenced by the activity of A(H3). The model is ready to integrate data from other pathogens that will become available in the near future.

## Competing Interests

The authors report no competing interests.

## References

[1] Hayward AC , Fragaszy EB , Bermingham A , et al. Comparative community burden and severity of seasonal and pandemic influenza: results of the Flu Watch cohort study. Lancet Respir Med. 2014;2:445–454. 24717637 10.1016/S2213-2600(14)70034-7 PMC7164821

[2] Aguilera JF , Paget WJ , Mosnier A , et al. Heterogeneous case definitions used for the surveillance of influenza in Europe. Eur J Epidemiol. 2003;18:751–754. 12974549 10.1023/a:1025337616327

[3] CDC (Centers for Disease Control and Prevention) . Overview of Influenza Surveillance in the United States. http://www.cdc.gov/flu/weekly/overview.htm. Accessed April 10, 2015.

[4] ECDC (European Centers for Disease Control and Prevention) . Influenza case definitions. http://ecdc.europa.eu/en/activities/surveillance/eisn/surveillance/pages/influenza_case_definitions.aspx. Accessed April 10, 2015.

[5] Carrat F , Vergu E , Ferguson NM , et al. Time lines of infection and disease in human influenza: a review of volunteer challenge studies. Am J Epidemiol. 2008;167:775–785. 18230677 10.1093/aje/kwm375

[6] Suess T , Remschmidt C , Schink SB , et al. Comparison of shedding characteristics of seasonal influenza virus (sub)types and influenza A(H1N1)pdm09; Germany, 2007‐2011. PLoS One. 2012;7:e51653. 23240050 10.1371/journal.pone.0051653 PMC3519848

[7] Loeb M , Singh PK , Fox J , et al. Longitudinal study of influenza molecular viral shedding in Hutterite communities. J Infect Dis. 2012;206:1078–1084. 22837493 10.1093/infdis/jis450

[8] Lau LL , Cowling BJ , Fang VJ , et al. Viral shedding and clinical illness in naturally acquired influenza virus infections. J Infect Dis. 2010;201:1509–1516. 20377412 10.1086/652241 PMC3060408

[9] Jiang L , Lee VJ , Lim WY , et al. Performance of case definitions for influenza surveillance. Euro Surveill. 2015;20:21145. 26062645 10.2807/1560-7917.es2015.20.22.21145

[10] an der Heiden . M, Köpke K, Buda S, Buchholz U, Haas W. Estimates of excess medically attended acute respiratory infections in periods of seasonal and pandemic influenza in Germany from 2001/02 to 2010/11. PLoS One. 2013;8:e64593. 23874380 10.1371/journal.pone.0064593 PMC3712969

[11] Arbeitsgemeinschaft Influenza . Bericht zur Epidemiologie der Influenza in Deutschland, Saison 2014/15. [Report on the Epidemiology of Influenza, Season 2014/15.] ISBN 978‐3‐89606‐265‐9. influenza.rki.de/agi > Saisonberichte. Accessed February 2, 2016.

[12] Wood SN . Generalized Additive Models: An introduction with R. Boca Raton, FL: Chapman & Hall/CRC; 2006 xvii, 391 p.

[13] R Core Team . R: A Language and Environment for Statistical Computing. Vienna, Austria: R Foundation for Statistical Computing; 2015 https://www.R-project.org.

[14] Paget WJ , Balderston C , Casas I , et al. Assessing the burden of paediatric influenza in Europe: the European Paediatric Influenza Analysis (EPIA) project. Eur J Pediatr. 2010;169:997–1008. 20229049 10.1007/s00431-010-1164-0 PMC2890072

[15] Poehling KA , Edwards KM , Griffin MR , et al. The burden of influenza in young children, 2004‐2009. Pediatrics. 2013;131:207–216. 23296444 10.1542/peds.2012-1255 PMC3557405

[16] Fowlkes A , Steffens A , Temte J , et al. Incidence of medically attended influenza during pandemic and post‐pandemic seasons through the Influenza Incidence Surveillance Project, 2009‐13. Lancet Respir Med. 2015;3:709–718. 26300111 10.1016/S2213-2600(15)00278-7 PMC5749913

[17] Goldstein E , Cobey S , Takahashi S , Miller JC , Lipsitch M . Predicting the epidemic sizes of influenza A/H1N1, A/H3N2, and B: a statistical method. PLoS Med. 2011;8:e1001051. 21750666 10.1371/journal.pmed.1001051 PMC3130020

[18] Wagner AP , McKenzie E , Robertson C , McMenamin J , Reynolds A , Murdoch H . Automated mortality monitoring in Scotland from 2009. Euro Surveill. 2013;18:20451. 23594577

[19] Cromer D , van Hoek AJ , Jit M , Edmunds WJ , Fleming D , Miller E . The burden of influenza in England by age and clinical risk group: a statistical analysis to inform vaccine policy. J Infect. 2014;68:363–371. 24291062 10.1016/j.jinf.2013.11.013

[20] Fleming DM , Zambon M , Bartelds AI . Population estimates of persons presenting to general practitioners with influenza‐like illness, 1987‐96: a study of the demography of influenza‐like illness in sentinel practice networks in England and Wales, and in The Netherlands. Epidemiol Infect. 2000;124:245–253. 10813150 10.1017/s0950268899003660 PMC2810908

[21] Monto AS , Koopman JS , Longini IM Jr . Tecumseh study of illness. XIII. Influenza infection and disease, 1976‐1981. Am J Epidemiol. 1985;121:811–822. 4014174 10.1093/oxfordjournals.aje.a114052

[22] Meijer A , Paget WJ , Meerhoff TJ , et al. Epidemiological and virological assessment of influenza activity in Europe, during the 2004‐2005 winter. Euro Surveill. 2006;11:111–118. 16757850

[23] Bodewes R , de Mutsert G , van der Klis FR , et al. Prevalence of antibodies against seasonal influenza A and B viruses in children in Netherlands. Clin Vaccine Immunol. 2011;18:469–476. 21209157 10.1128/CVI.00396-10 PMC3067385

[24] Sauerbrei A , Langenhan T , Brandstadt A , et al. Prevalence of antibodies against influenza A and B viruses in children in Germany, 2008 to 2010. Euro Surveill. 2014;19(5). 10.2807/1560-7917.es2014.19.5.20687 24524235

[25] du Prel JB , Puppe W , Grondahl B , et al. Are meteorological parameters associated with acute respiratory tract infections? Clin Infect Dis. 2009;49:861–868. 19663691 10.1086/605435

