# Supplementary material for: Estimation of influenza‐attributable medically attended acute respiratory illness by influenza type/subtype and age, Germany, 2001/02–2014/15
Source: Influenza Other Respir Viruses. 2016 Nov 18;11(2):110–21. doi: 10.1111/irv.12434 (PMC5304576; doi:10.1111/irv.12434)
Supplement: Supplementary file 1 [file IRV-11-110-s001.zip › S4_comp_baseline_0708.pdf]

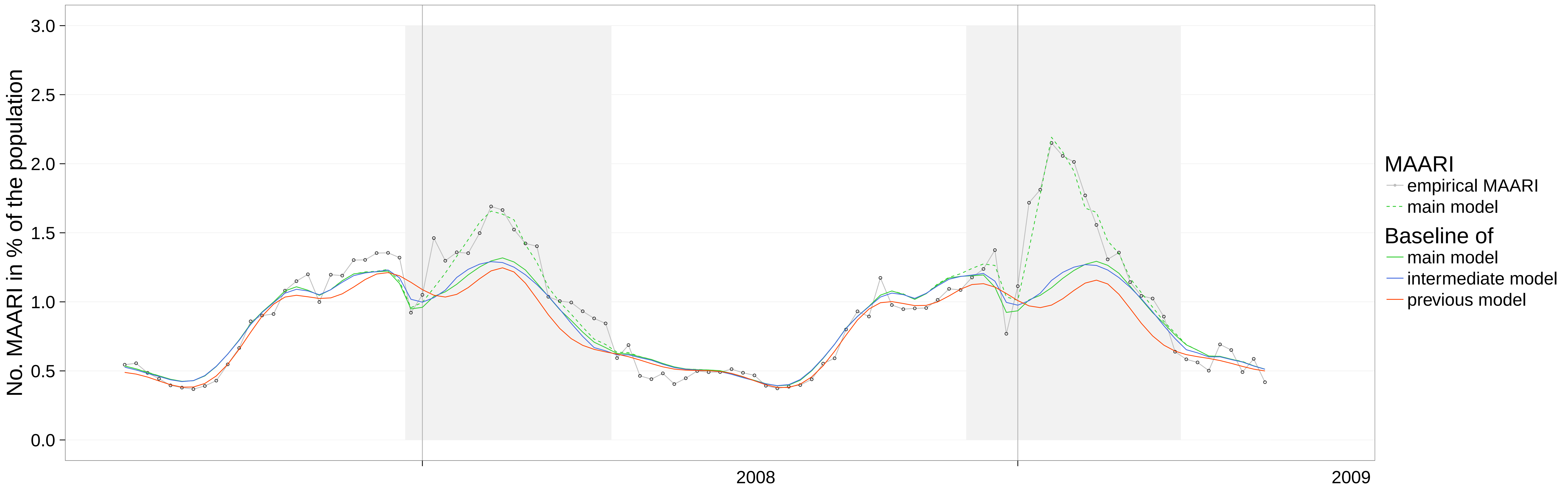
No. MAARI in % of the population
3.0
2.5
2.0
1.5
1.0
0.5
0.0
2008
2009
MAARI
empirical MAARI
main model
Baseline of
main model
intermediate model
previous model
