# Supplementary figures and images for: Estimation of influenza‐attributable medically attended acute respiratory illness by influenza type/subtype and age, Germany, 2001/02–2014/15
Source: Influenza Other Respir Viruses. 2016 Nov 18;11(2):110–21. doi: 10.1111/irv.12434 (PMC5304576; doi:10.1111/irv.12434)

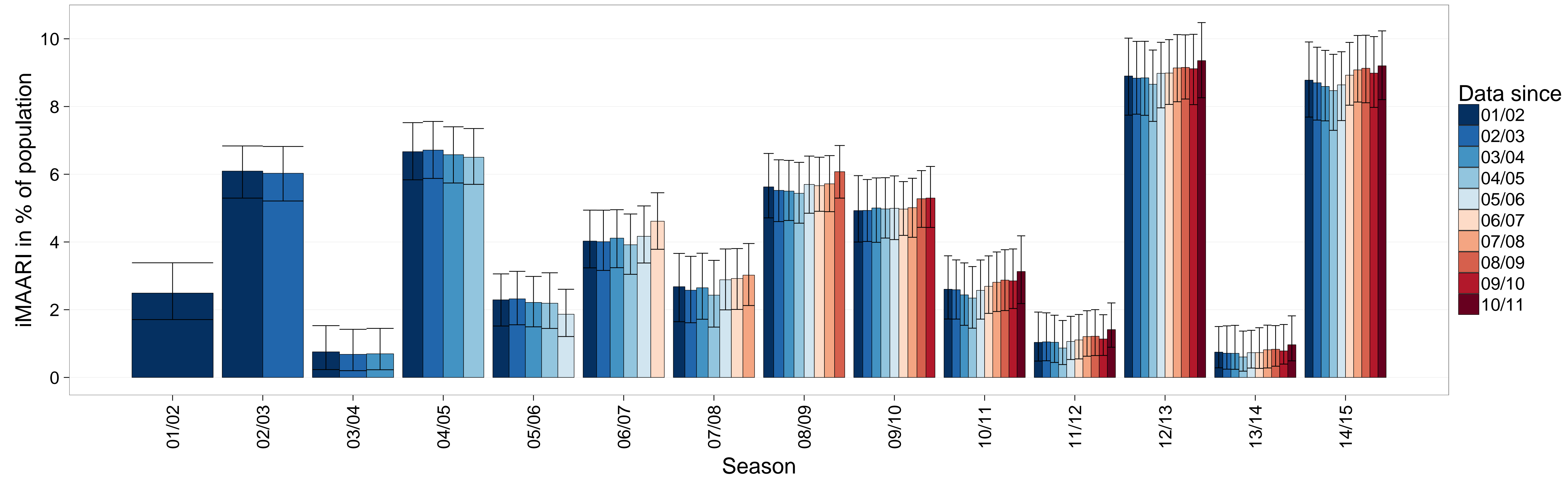

Supplement: Supplementary file 1 [file IRV-11-110-s001.zip › S1_main.pdf]

iMAARI in % of the population

0–4 years old

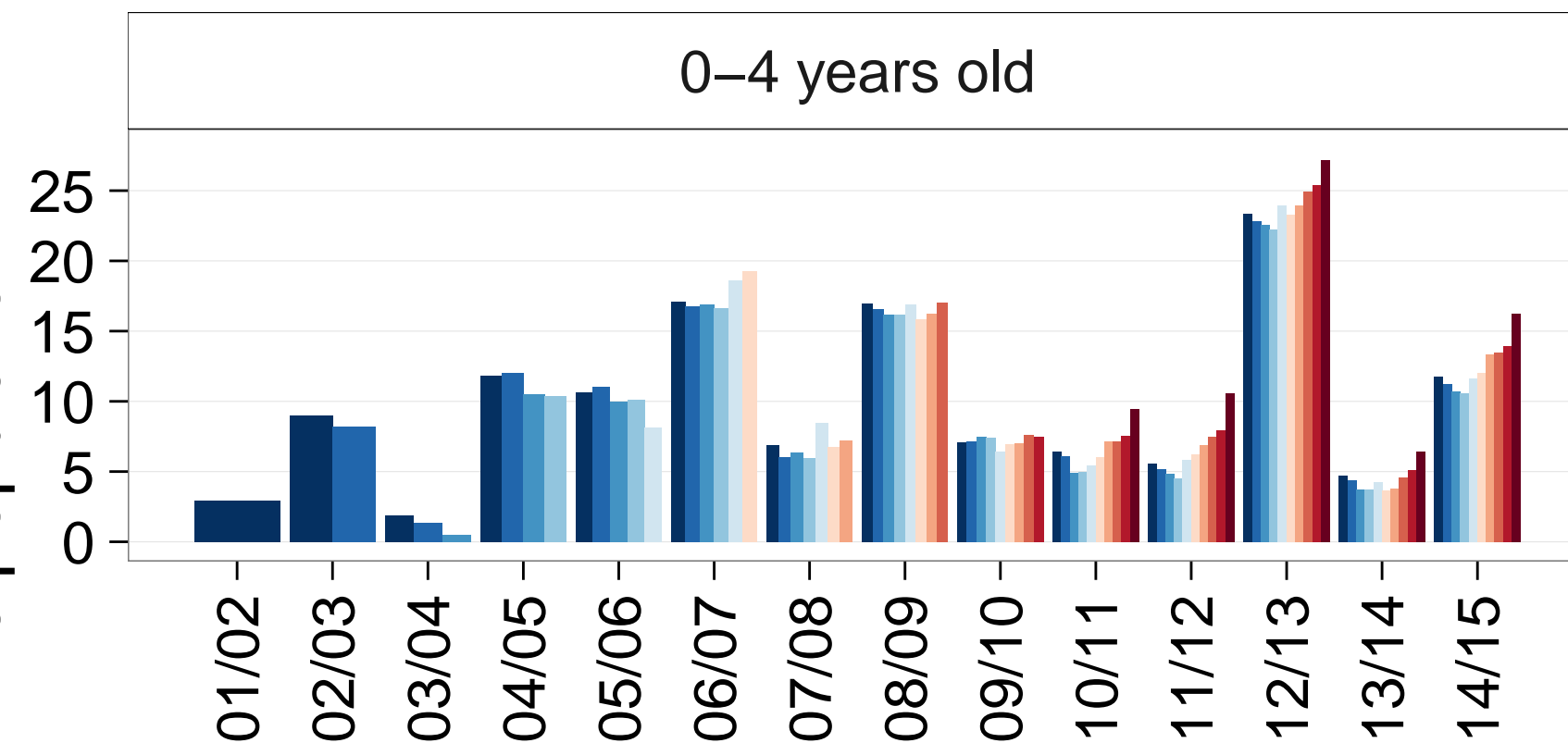

5–14 years old

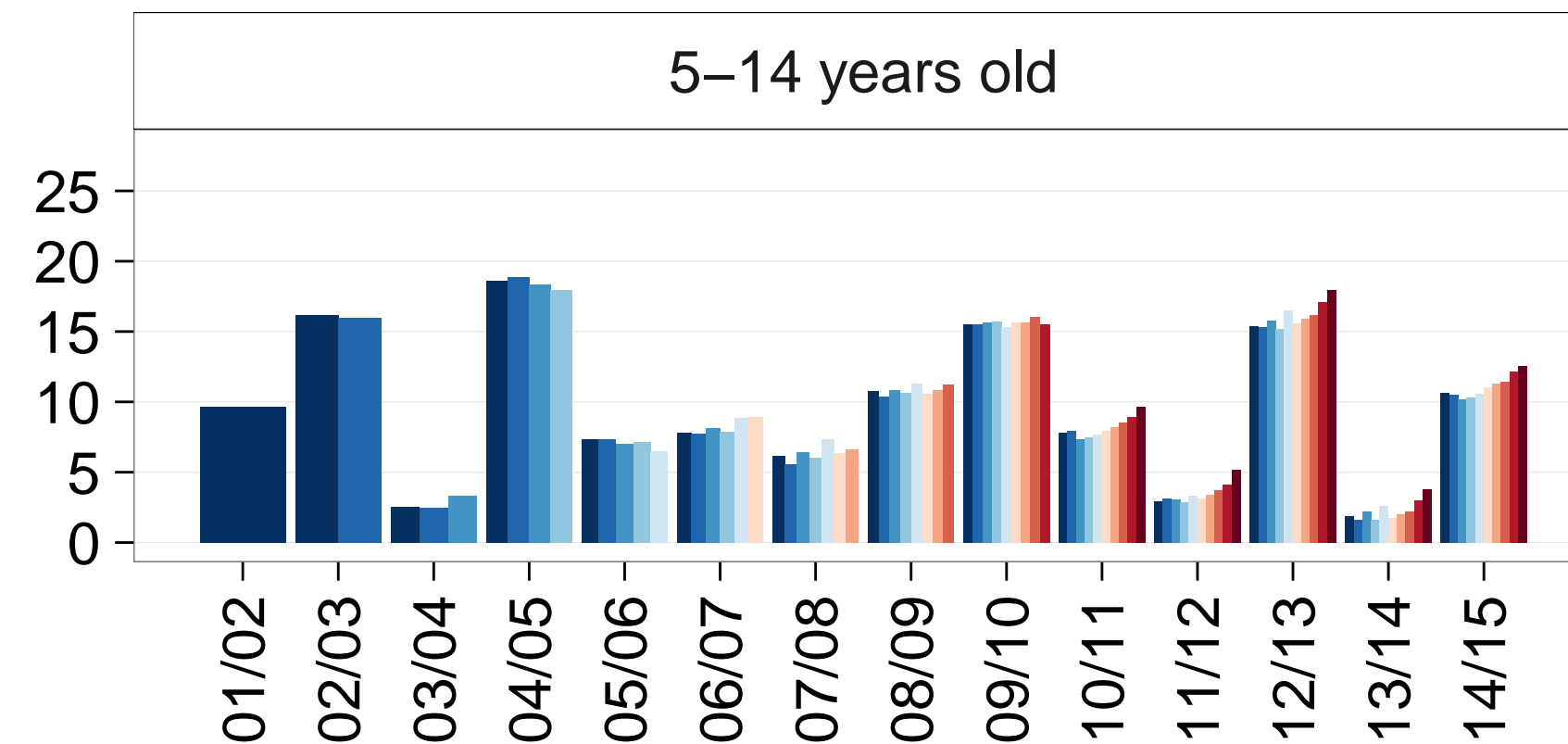

15–34 years old

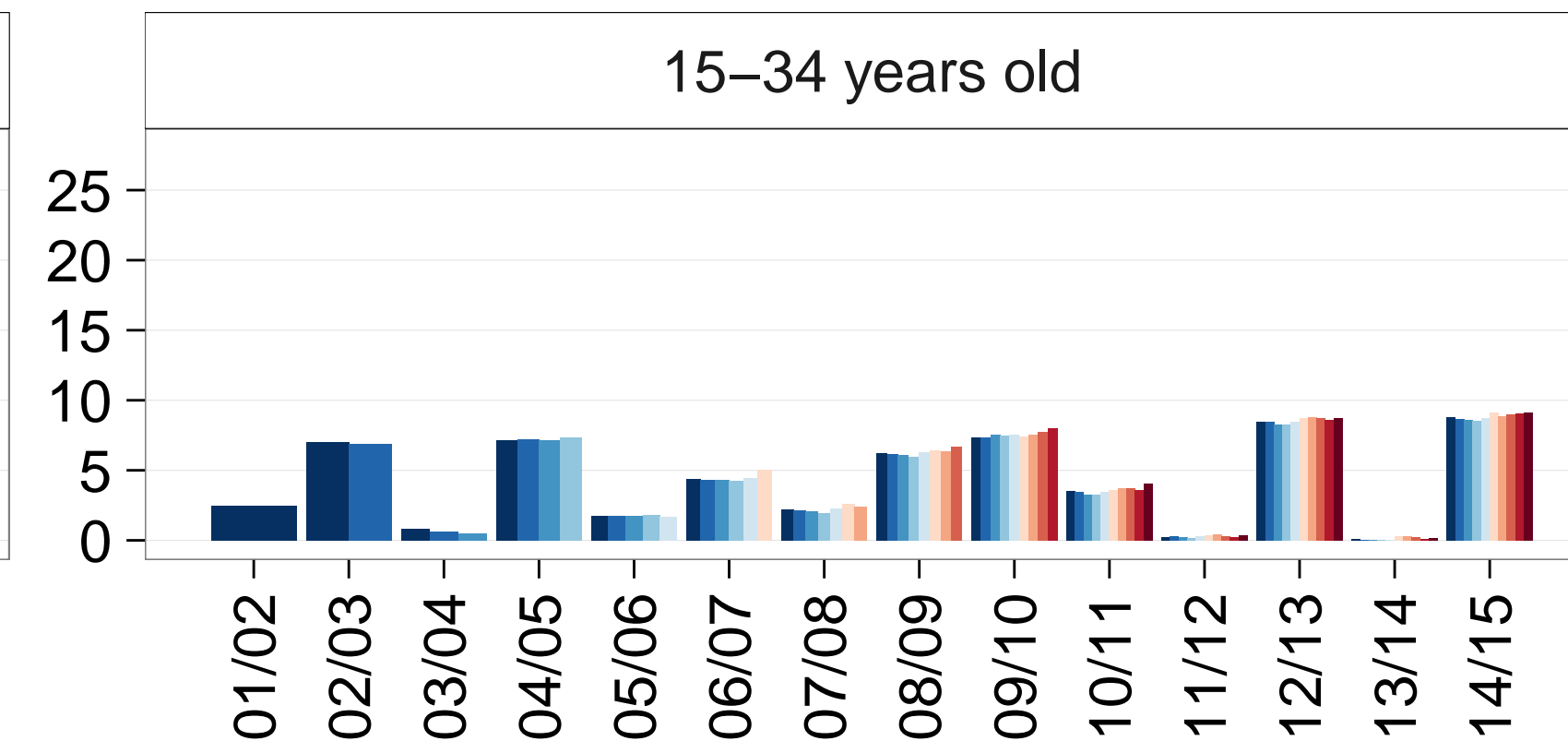

35–59 years old

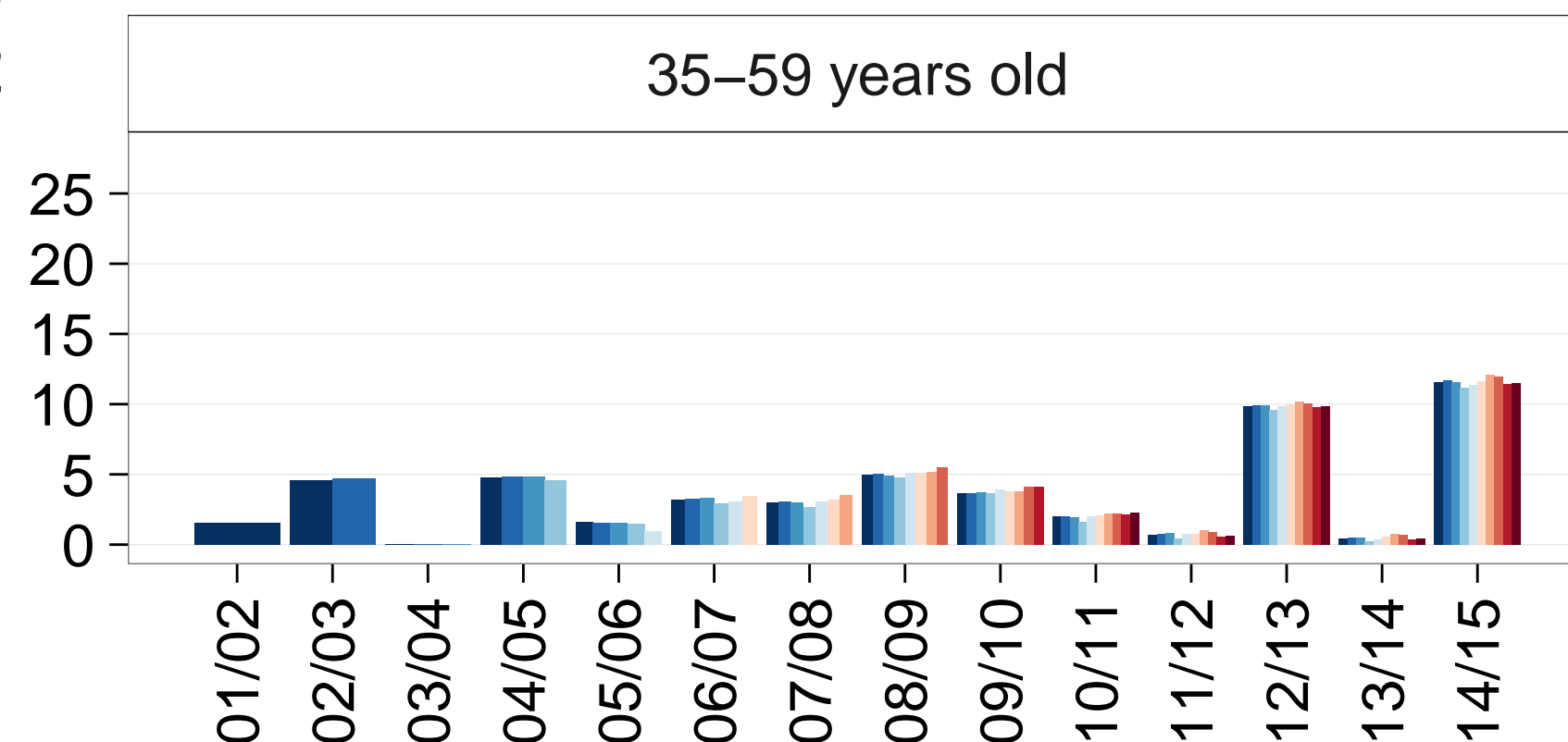

60+ years old

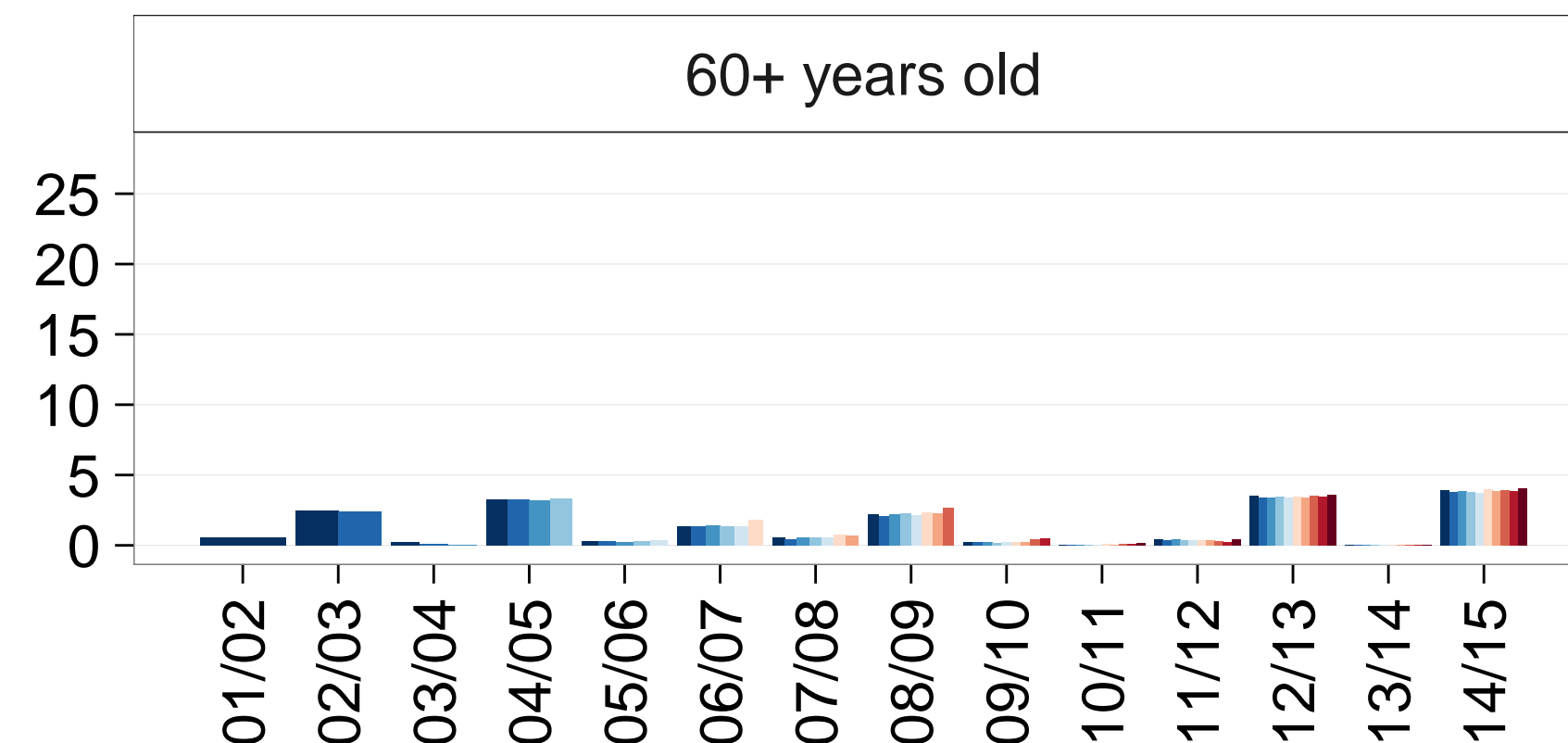

Data since

01/02  
02/03  
03/04  
04/05  
05/06  
06/07  
07/08  
08/09  
09/10  
10/11

Supplement: Supplementary file 1 [file IRV-11-110-s001.zip › S1_mainAG.pdf]

iMAARI in % of the population

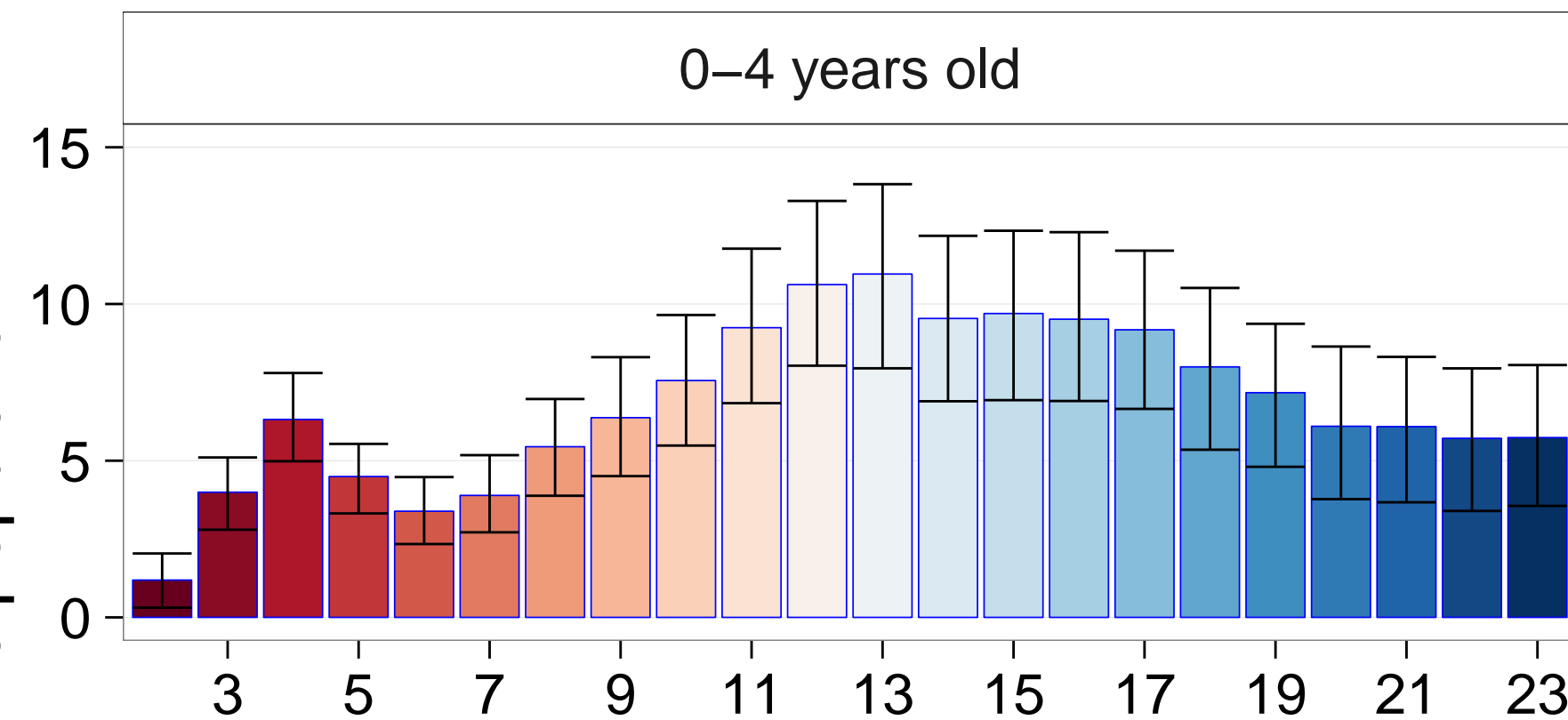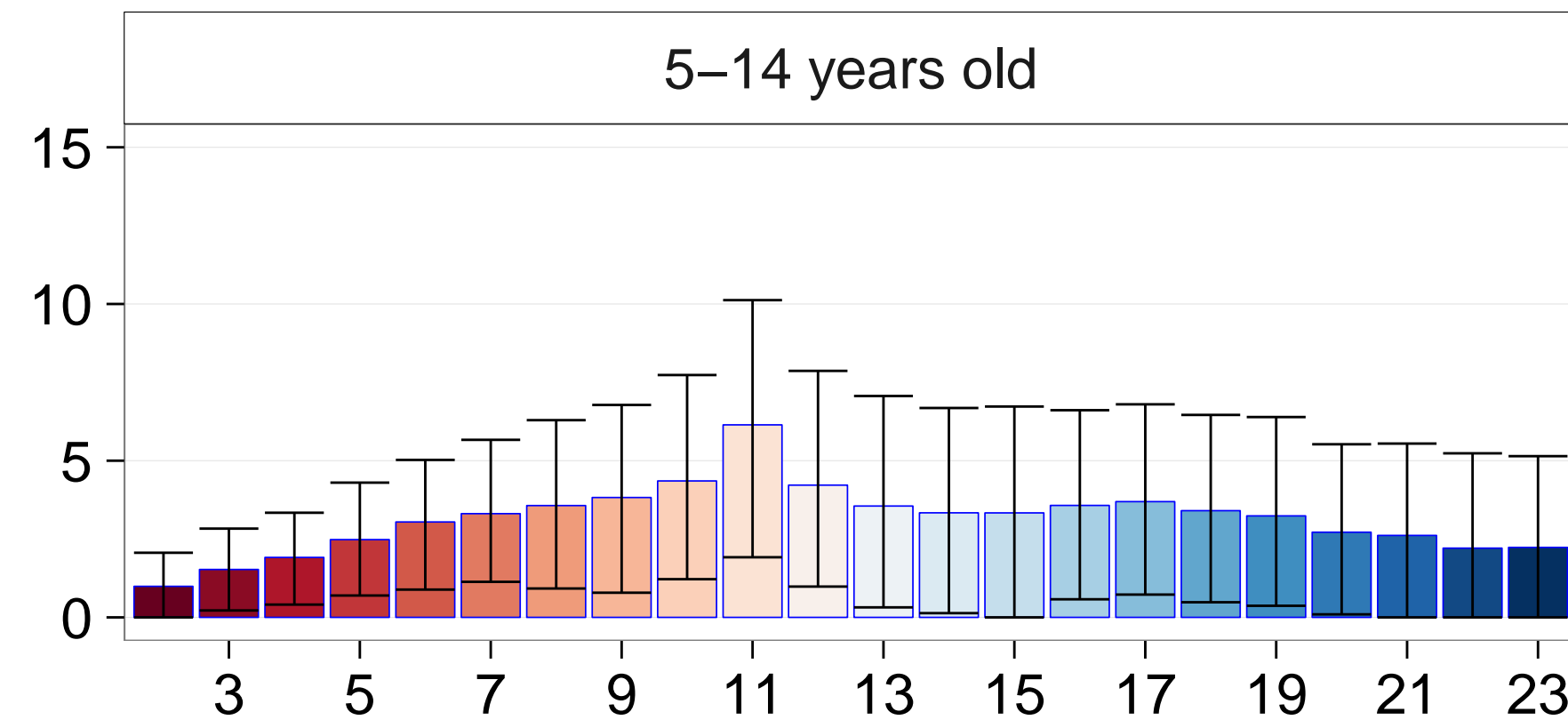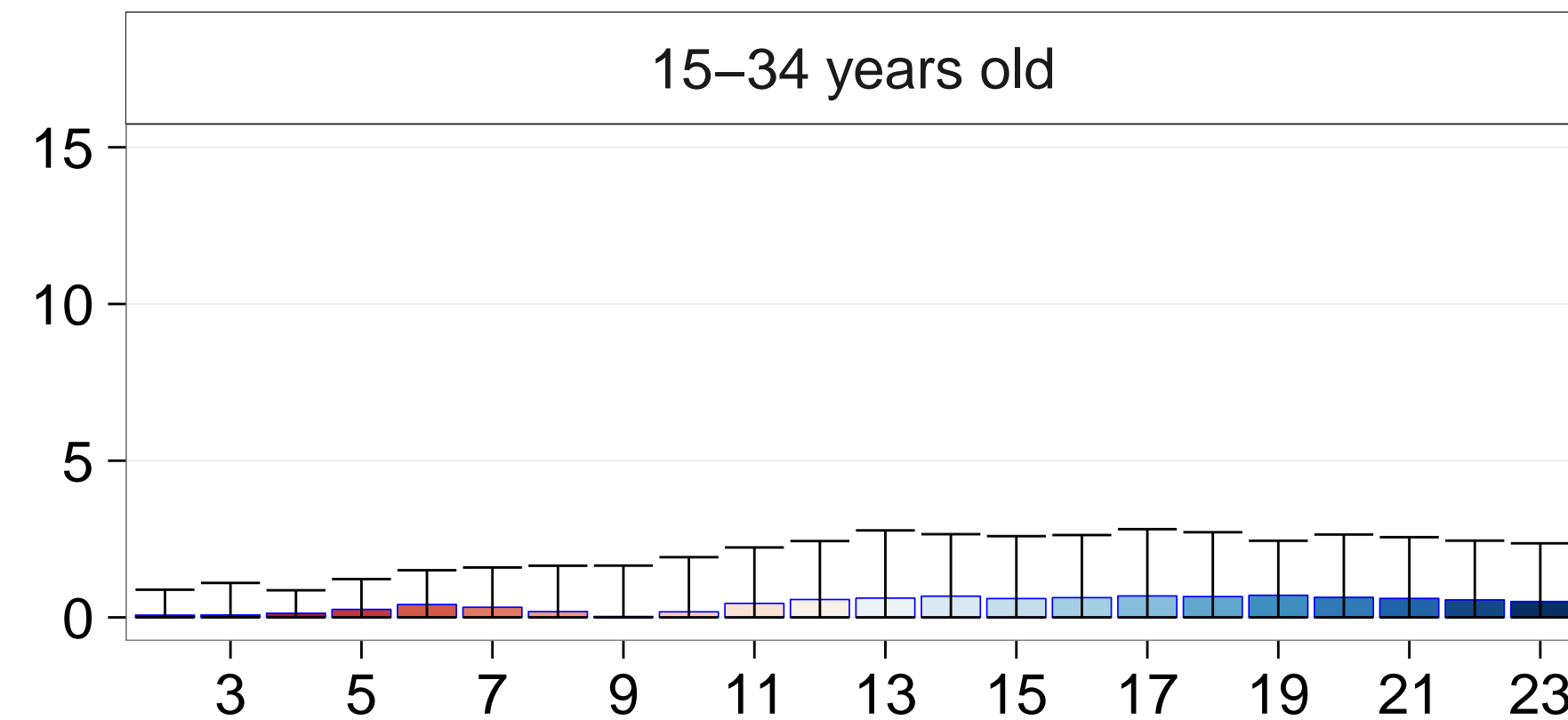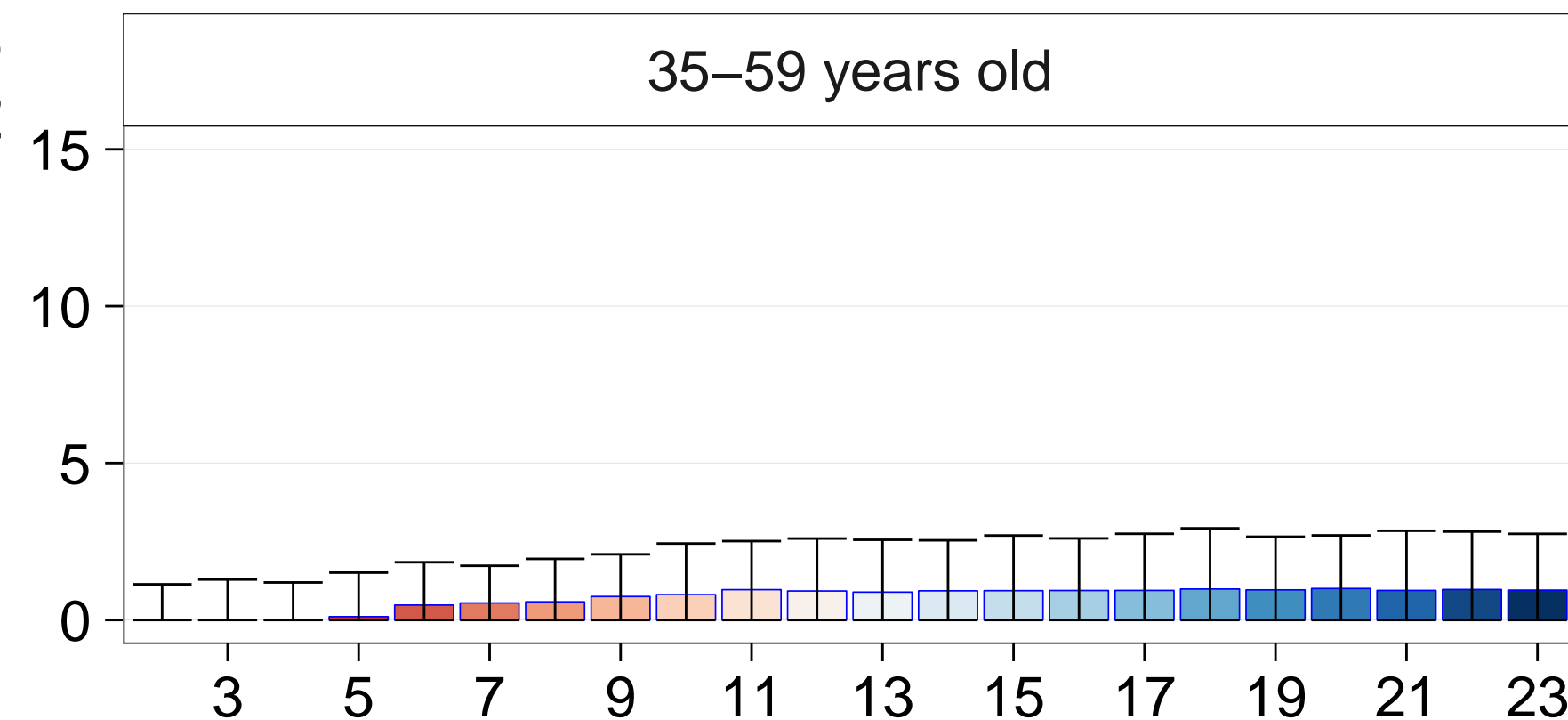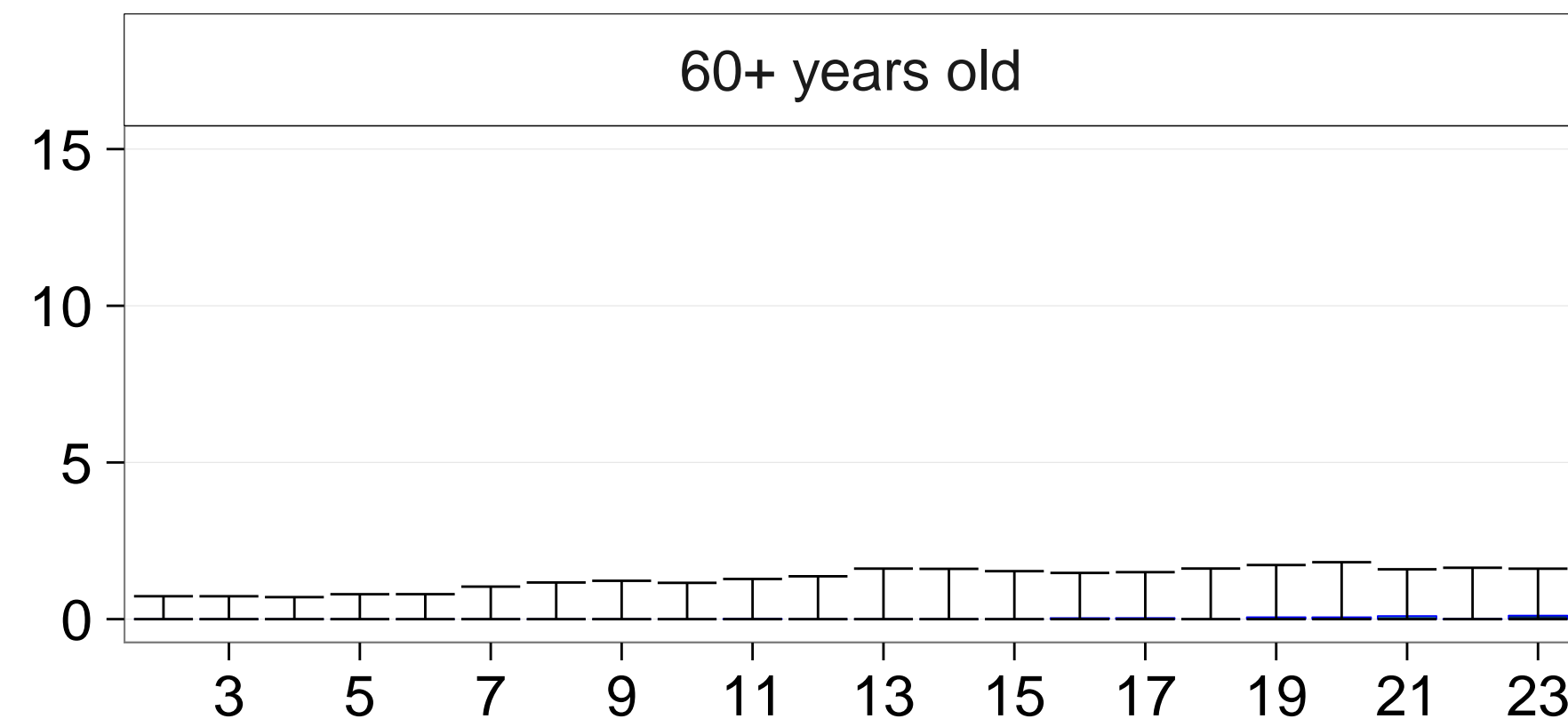

Calendar week in 2014

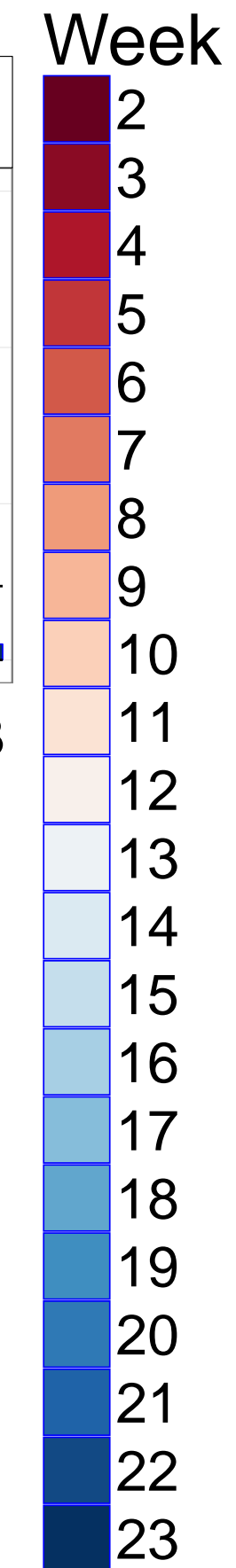

Supplement: Supplementary file 1 [file IRV-11-110-s001.zip › S2_flex_onlineAG_2013.pdf]

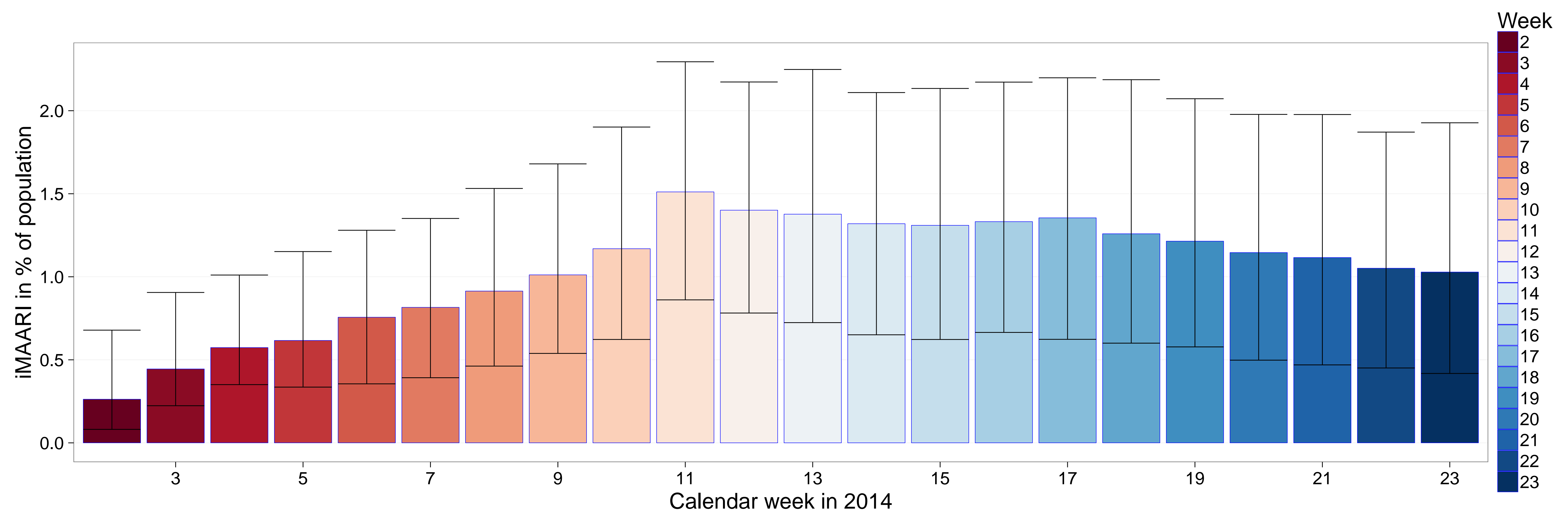

Supplement: Supplementary file 1 [file IRV-11-110-s001.zip › S2_flex_online_2013.pdf]

iMAARI in % of the population

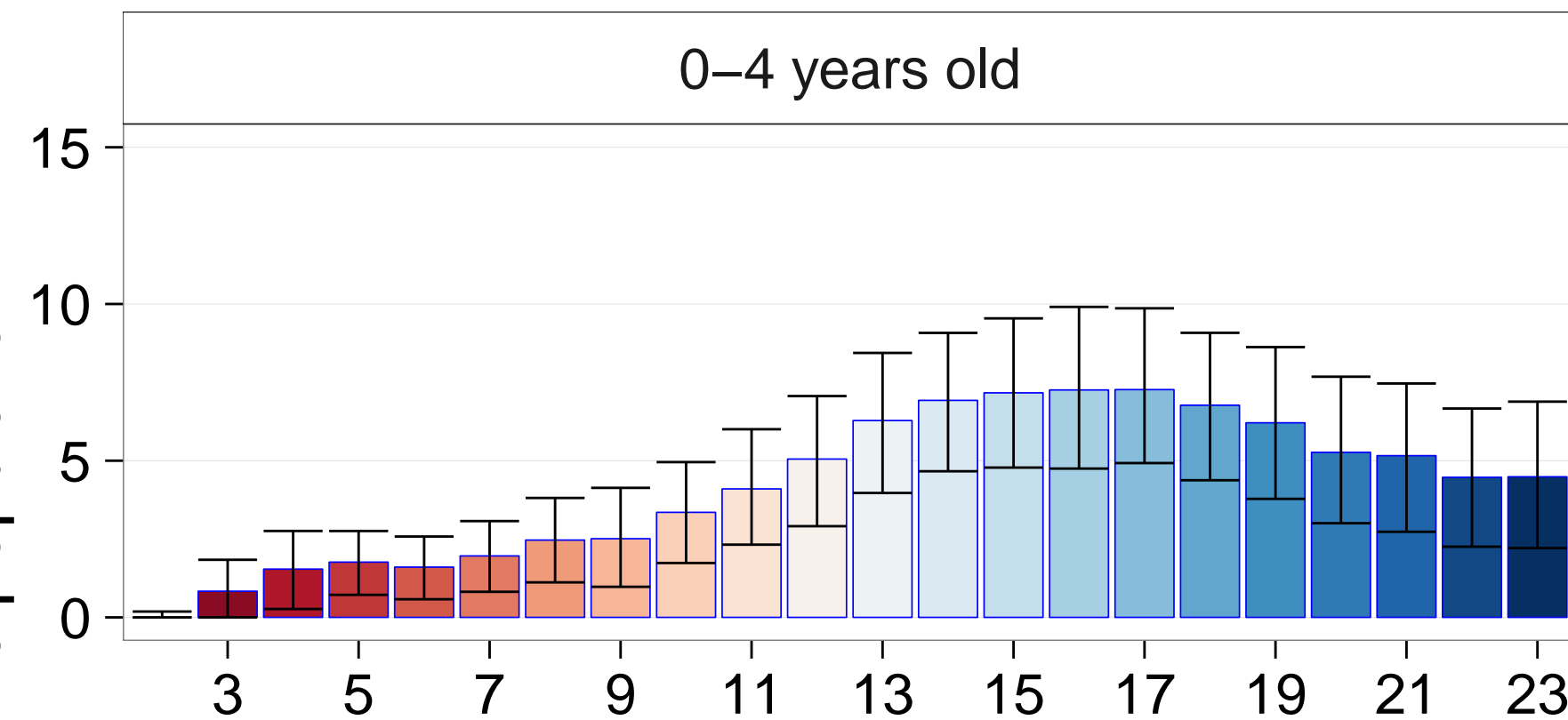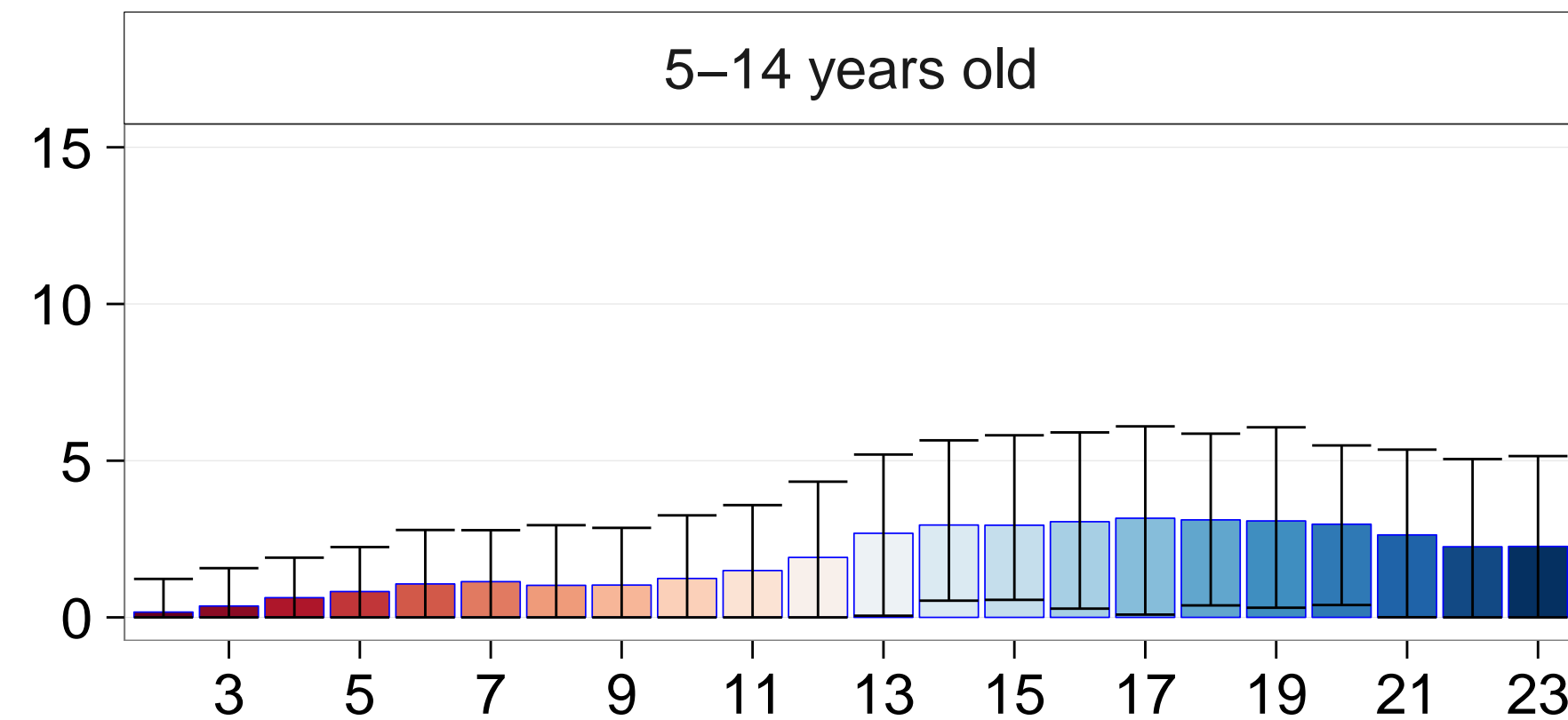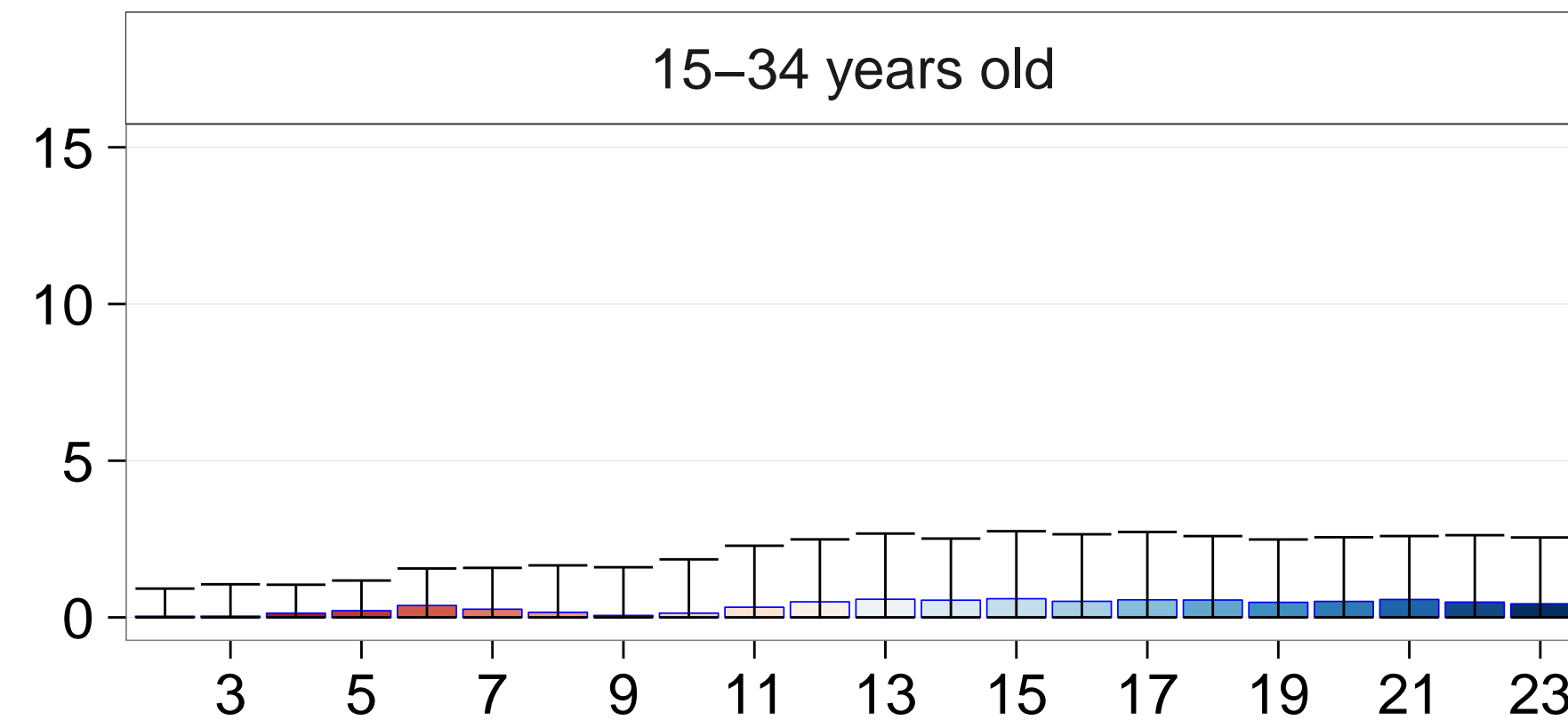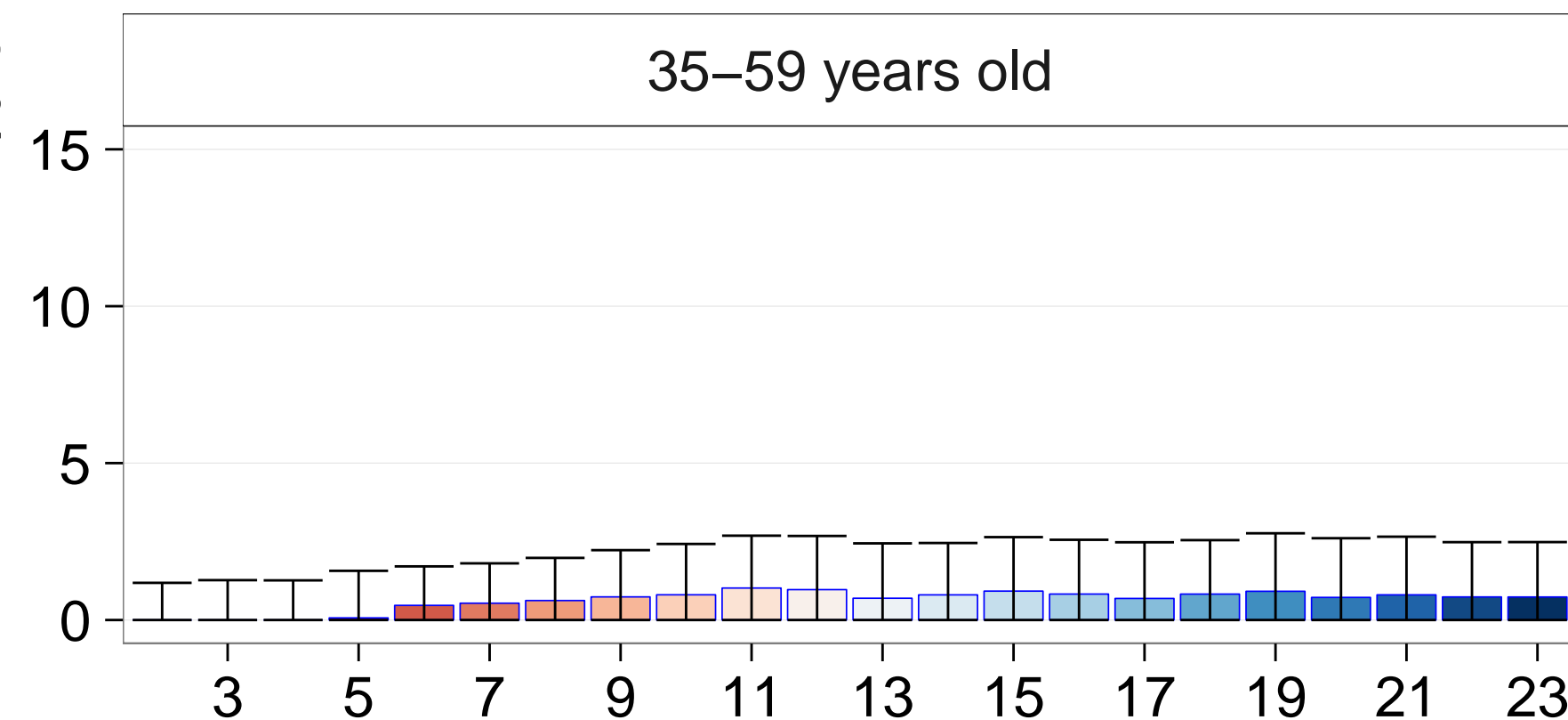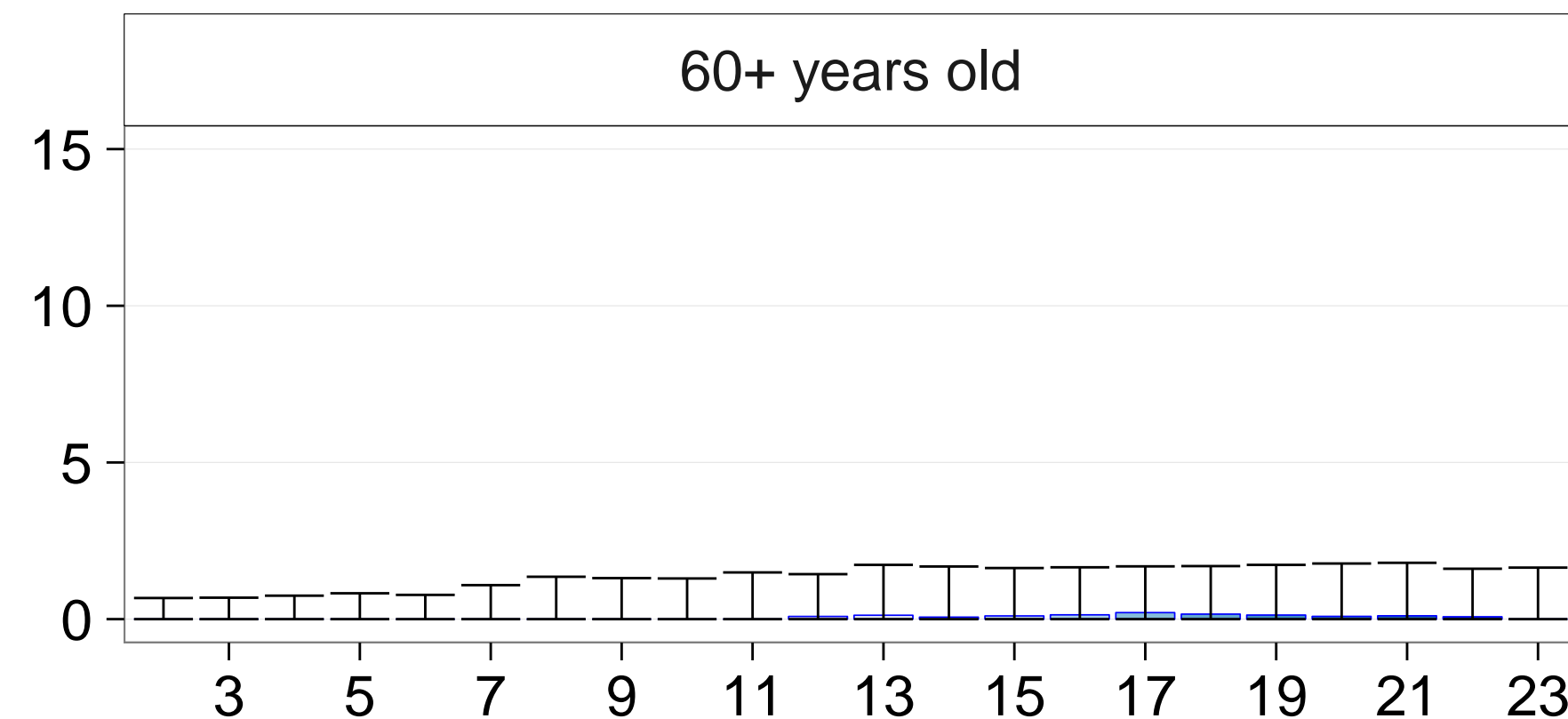

Calendar week in 2015

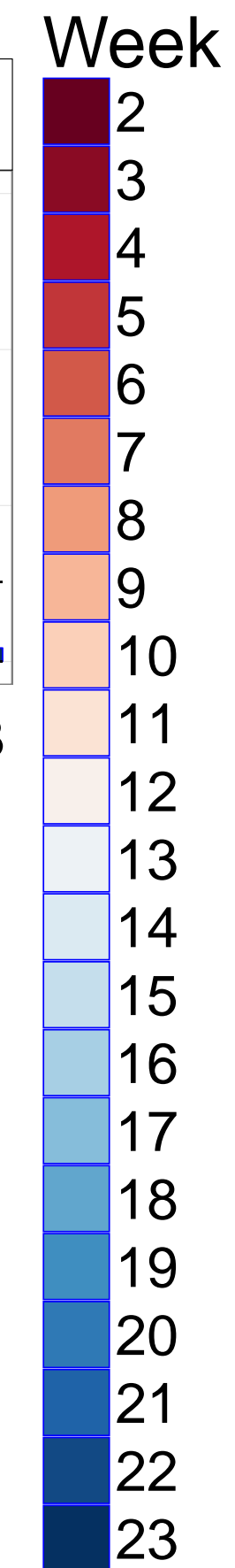

Supplement: Supplementary file 1 [file IRV-11-110-s001.zip › S2_main_onlineAG_2013.pdf]

iMAARI in % of the population

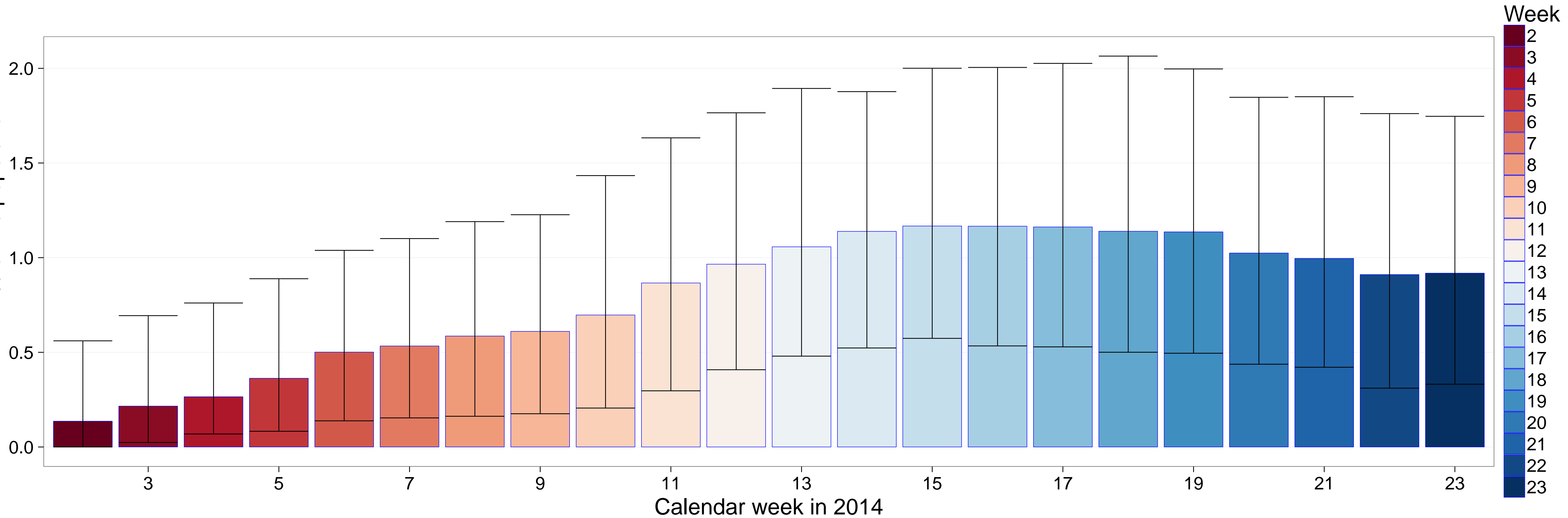

Supplement: Supplementary file 1 [file IRV-11-110-s001.zip › S2_main_online_2013.pdf]

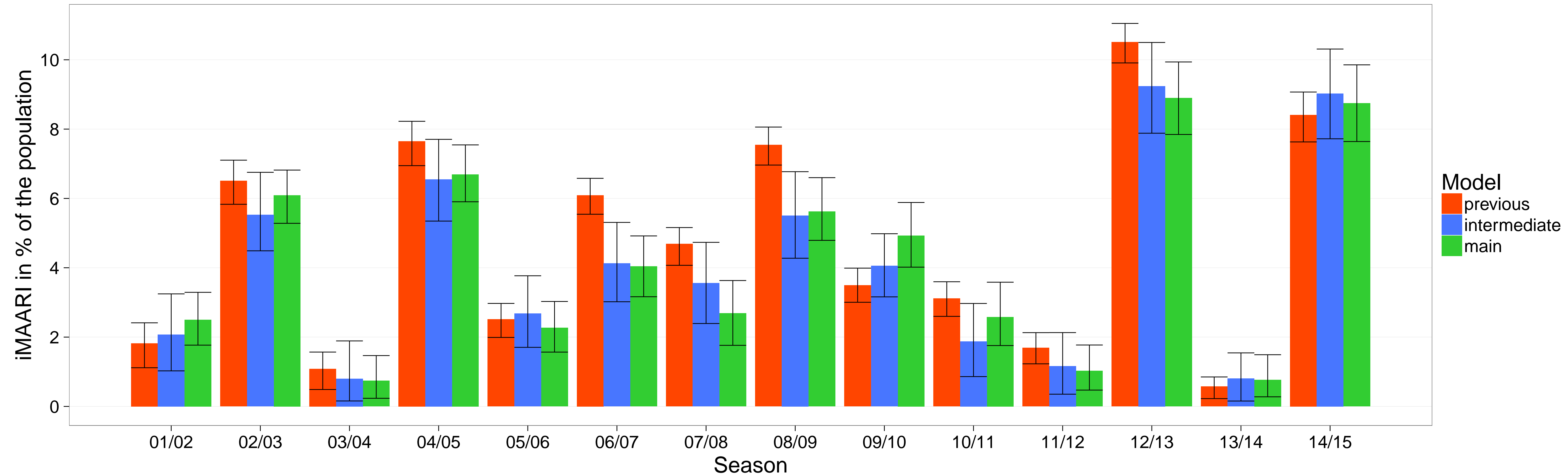

Supplement: Supplementary file 1 [file IRV-11-110-s001.zip › S3_Exc_Comp.pdf]

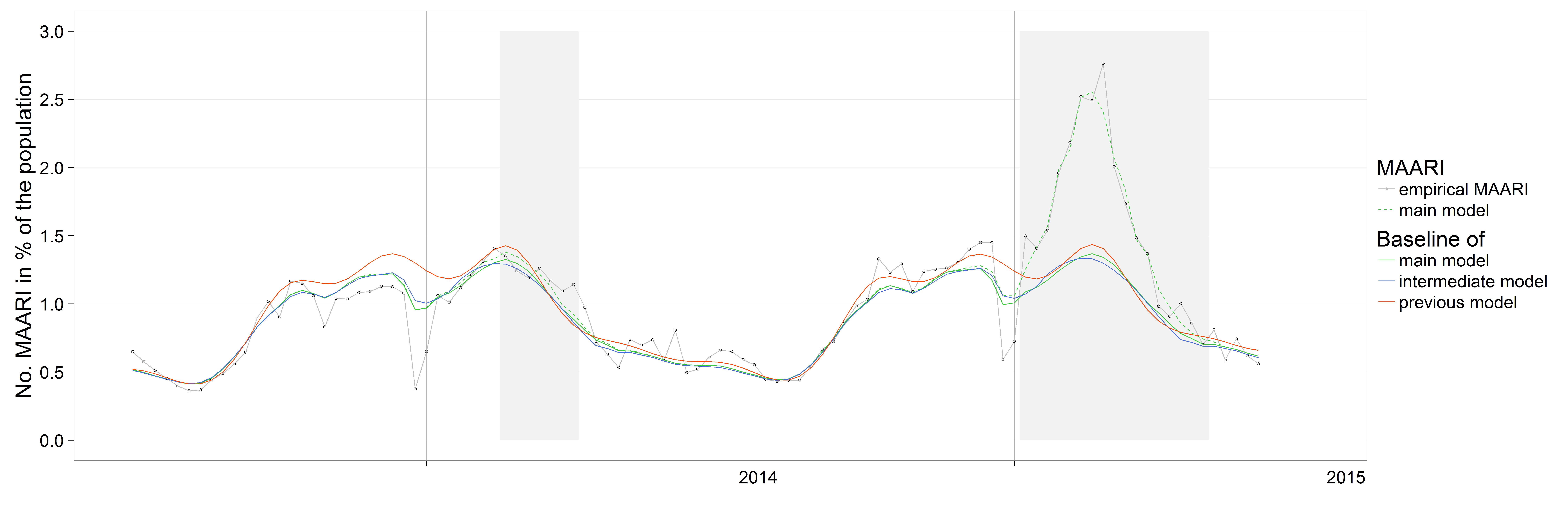

Supplement: Supplementary file 1 [file IRV-11-110-s001.zip › S4_comp_baseline_1314.jpg]
